# Supramolecular self-associating amphiphiles (SSAs) as nanoscale enhancers of cisplatin anticancer activity†

**DOI:** 10.1039/d1ra02281d

**Published:** 2021-04-15

**Authors:** Nova O. Dora, Edith Blackburn, Jessica E. Boles, George T. Williams, Lisa J. White, Scarlett E. G. Turner, J. Daniel Hothersall, Trevor Askwith, Jack A. Doolan, Daniel P. Mulvihill, Michelle D. Garrett, Jennifer R. Hiscock

**Affiliations:** School of Biosciences, University of Kent Canterbury Kent CT2 7NJ UK M.D.Garrett@Kent.ac.uk; School of Physical Sciences, University of Kent Canterbury Kent CT2 7NH UK J.R.Hiscock@Kent.ac.uk; School of Chemistry, University of Birmingham Edgbaston Birmingham West Midlands B15 2TT UK; Domainex, Chesterford Research Park Saffron Walden CB10 1XL UK

## Abstract

Many chemotherapeutic drugs have a narrow therapeutic window due to inefficient tumour cell permeation. Supramolecular self-associating amphiphilic salts (SSAs) are a unique class of small molecules that offer potential as next generation cancer drugs and/or therapeutic enhancement agents. Herein, we demonstrate the cytotoxicity of seven SSAs towards both ovarian and glioblastoma cancer cells. We also utilize the intrinsic fluorescent properties of one of these lead SSAs to provide evidence for this class of compound to both bind to the exterior cancer cell surface and permeate the cell membrane, to become internalized. Furthermore, we demonstrate synergistic effects of two lead SSAs on cisplatin-mediated cytotoxicity of ovarian cancer cells and show that this correlates with increased DNA damage and apoptosis *versus* either agent alone. This work provides the first evidence that SSAs interact with and permeate cancer cell membranes and enhance the cytotoxic activity of a chemotherapeutic drug in human cancer cells.

## Abstract 2

Herein we show the potential SSA technology towards the development of synergistic next-generation anticancer treatments.

## Graphical abstract

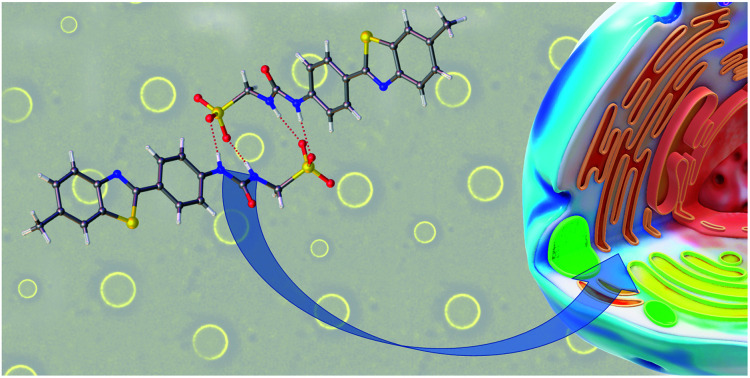

## Introduction

Cancer is a major global health problem; it is the second highest cause of death worldwide, resulting in almost 9.9 million deaths in 2020.^1^ Owing to the challenges inherent in designing diseased cell specific treatments, many marketed drugs cause toxicity towards healthy cells.^2^ This leads to a myriad of adverse health effects for the patient, including early mortality.^3^ However, cytotoxic chemotherapies, such as cisplatin remain one of the most effective therapeutic strategies for a range of cancers, including ovarian cancer.^4^ However, the use and dosage of these therapies are limited by nephrotoxicity and both intrinsic and acquired tumor resistance to drug action.^5^ Therefore, the development of agents to selectively enhance the efficacy of these cytotoxic chemotherapies and overcome cellular resistance mechanisms, thus lowering the effective doses of a chemotherapy to be administered is of the utmost importance.

**Fig. 1:**
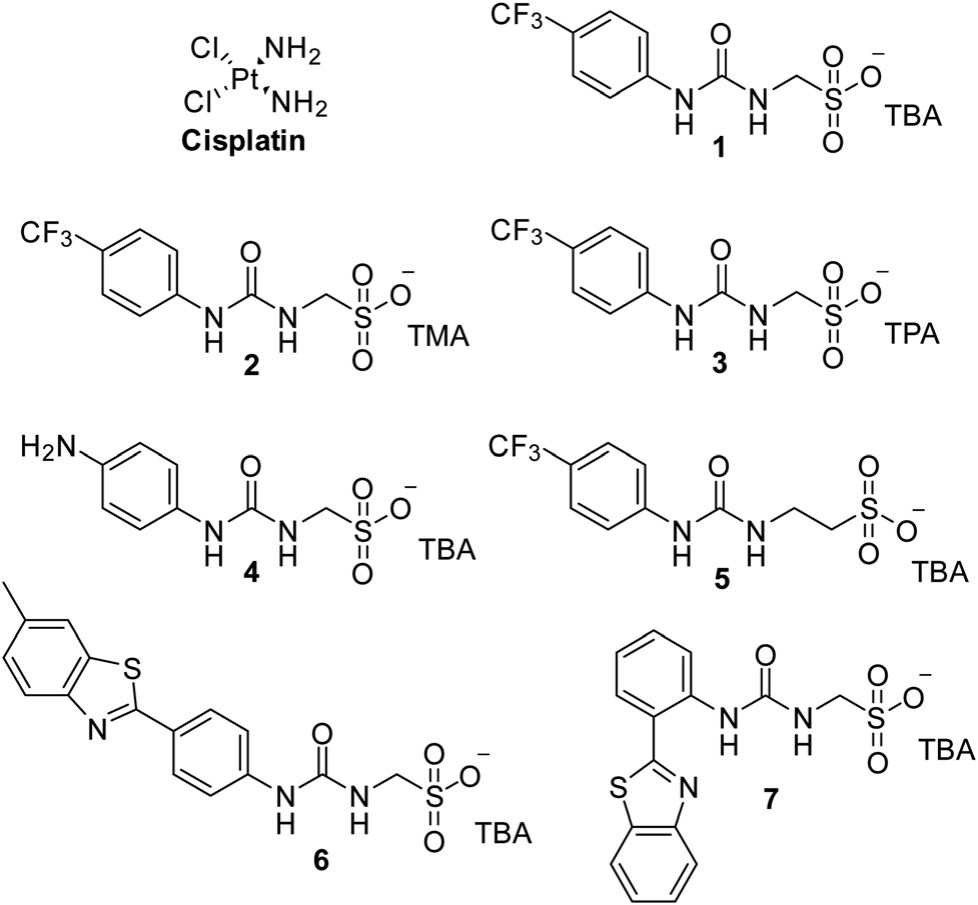
Chemical structure of cisplatin and SSAs 1–7. TBA = tetrabutylammonium, TMA = tetramethylammonium, TPA = tetrapropylammonium.

**Fig. 2:**
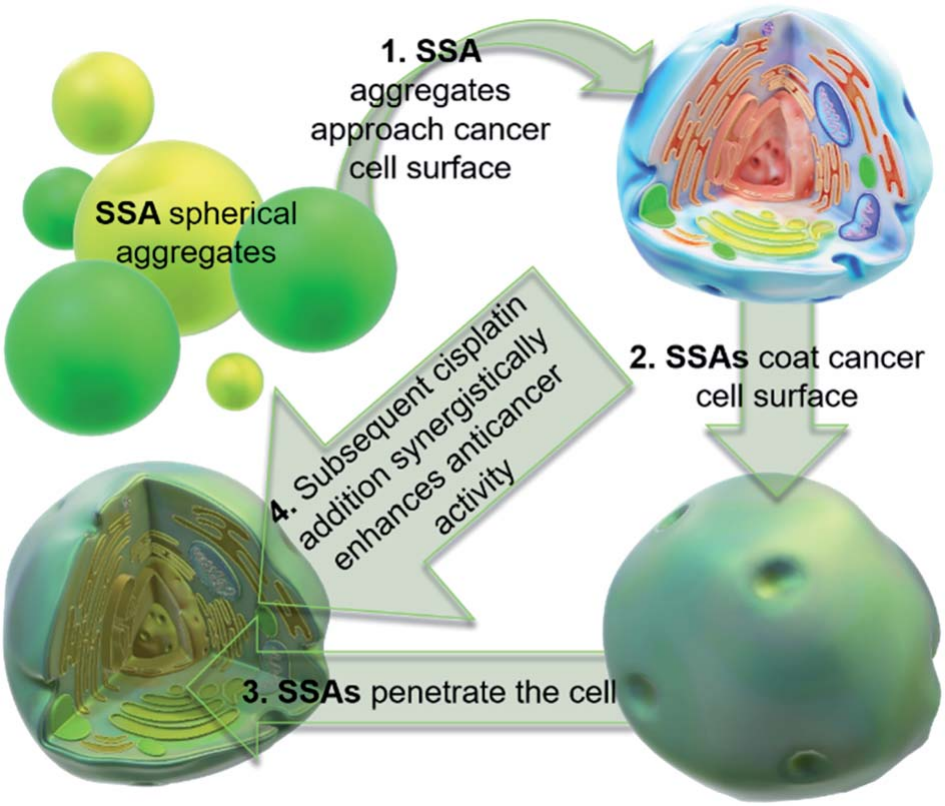
Cartoon illustrating the hypothesised mode of SSA anticancer activity, where the SSAs arrive at the cancer cell surface as self-associated spherical aggregates (evidence provided in previously published data).^8,9^ The SSAs initially interact with the external cell surface to form a coating and are then internalised (evidence provided in Fig. 3). This process enhances the efficacy of the cancer drug cisplatin, upon addition after prior incubation of the cells with an SSA (evidence provided in Fig. 4 and 5).

Fig. 1 shows the structures of seven (1–7) supramolecular self-associating amphiphilic salts (SSAs) which are members from a current ≈70 compound library.^6–8^ We have previously shown SSAs to: adopt a variety of environment dependent nanoscale structures; act as antimicrobial agents; act as antibiotic delivery materials and;^8,9^ act as antimicrobial efficacy enhancers.^10^ Based on these initial studies, we proposed the following SSA mode of antimicrobial action: SSAs arrive at the cell surface in a self-associated form (typically as spherical aggregates with a hydrodynamic diameter ≈ 100–550 nm in diameter), the monomeric constituents of which then interact selectively with the polar phospholipids present on the exterior bacterial surface such as phosphatidylethanolamine (PE),^11,12^ a process which causes membrane disruption/SSA internalization, resulting in an inhibition of bacterial growth. In healthy human cells these polar phospholipids are more commonly confined to the inner leaflet of the cellular membrane, while neutral (*e.g.* phosphatidylcholine) and zwitterionic (*e.g.* sphingomyelin) phospholipids are found at the extracellular cell surface.^13^ This asymmetric phospholipid distribution is maintained through the action of flippase enzymes.^14^ However, the activity of this enzyme in cancer cells is reduced, resulting in increased expression of phospholipids such as PE at the extracellular cell surface.^15^ Therefore, as illustrated in Fig. 2, we hypothesise that the mode of SSA antimicrobial activity could be mirrored in cancer cells, leading to the identification of SSAs as a potential novel cancer therapy and/or as molecular enhancers of cancer drug, in this case cisplatin, activity.

## Results and discussion

The GI_50_ values (compound concentration required to reduce the cellular growth by 50% *versus* untreated cells) for SSAs 1–7 and cisplatin against A2780 ovarian carcinoma and U87MG glioblastoma cell lines, are given in Table 1. In all cases, the GI_50_ values obtained were shown to be lower for the A2780 *versus* the U87MG cell line. Of the seven SSAs, 1 and 6 were shown to be the most effective against both cell lines. However, comparison of these GI_50_ values with that of cisplatin shows these SSAs to be several orders of magnitude less effective.

**Table 1:** GI_50_ values (μM) for cisplatin and 1–7 as determined by 96 hour sulforhodamine B (SRB)^16^ assay in A2780 and U87MG human cancer cell lines. Error = standard deviation of the mean

Compound	A2780	U87MG
Cisplatin	0.79 ± 0.4	6.41 ± 4.7
1	44.6 ± 19	248 ± 22
2	278 ± 18	395 ± 100
3	451 ± 38	>500
4	51.3 ± 17	297 ± 92
5	53.6 ± 17	316 ± 49
6	29.6 ± 9.7	189 ± 52
7	59.5 ± 8.1	367 ± 49

**Fig. 3:**
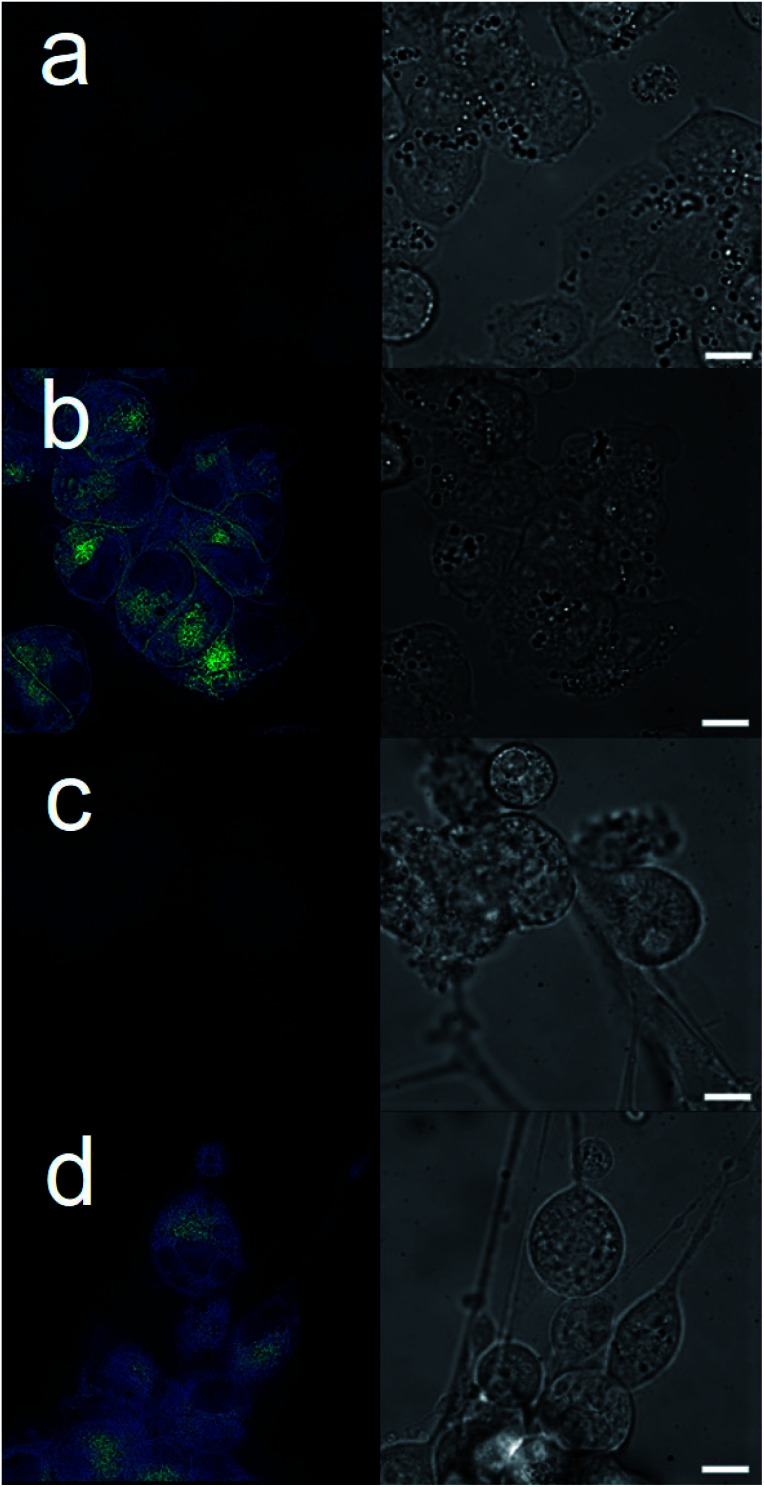
Live cell imaging fluorescence microscopy images of: (a) untreated A2780 cells; (b) A2780 cells after addition of 6 (90 μM); (c) untreated U87MG cells; (d) U87MG cells after addition of 6 (600 μM). Scale bar represents 10 μm.

The intrinsic fluorescent nature of 6 enabled live cell fluorescence microscopy experiments, which confirmed this SSA to both coordinate to the exterior cell surface and to become internalized within both A2780 and U87MG cell lines (Fig. 3). These results are analogous to those obtained for similar studies performed with 6 in the presence of bacteria.^8^ Moreover, these results support the hypothesis that SSAs have the potential to enhance permeability of the cancer cell membrane towards a subsequently administered chemotherapeutic agent, synergistically increasing anticancer efficacy. To verify this, two lead SSAs (1 and 6) identified from the GI_50_ studies (Table 1), were chosen to investigate any potential SSA:cisplatin synergistic anticancer effect against the A2780 cell line, to which the greatest SSA anticancer activity was demonstrated.

As 1 and 6 were to undergo investigation towards co-treatment applications with cisplatin, a range of physicochemical SSA : cisplatin 1 : 1 co-formulation studies were performed and confirmed that any resultant co-formulated self-associated nanoscale aggregates retained a similar size and stability to those of SSAs previously reported.^7,17^

An overview of results obtained from these studies can be found in Table 2. For a detailed discussion of the effects of cisplatin addition to the self-associated aggregates formed with 1 at 298 K in a H_2_O (or D_2_O)/5% ethanol solution, please see our previous work in this area.^10^ However, interestingly although the addition of cisplatin increases the proportion of the SSA to become incorporated into the larger self-associated structures of both 1 and 6 (quantitative ^1^H NMR), it has opposite effects on the size (hydrodynamic diameter – *D*_H_, obtained from DLS measurements), stability (obtained from zeta potential measurements) and critical micelle concentration (CMC) values, when compared to the values obtained for 1 and 6 alone. In summary the self-associated aggregate structures obtained with 1 increase in size and are destabilised upon the addition of cisplatin, however, the self-associated aggregate structures obtained with 6 decrease in size and are stabilised upon the addition of cisplatin. We hypothesise that this is due to the differences in the substituted phenyl ring systems of these two SSAs, which alter the way in which the anionic component of 1 and 6 interact with the cisplatin co-formulant during self-associative aggregate formation. It is also possible that these SSA, cisplatin interactions are in-part responsible for any symbiotic enhancement in anti-cancer activity.

**Table 2:** Where an SSA and cisplatin were present as a co-formulation, they have been supplied in a 1 : 1 molar ratio. For processed data please see Fig. S7–S25 (see ESI). CMC, DLS and zeta potential data were obtained for a H_2_O/5.0% EtOH solutions of an SSA or 1 : 1 cisplatin co-formulation (0.56 mM) at 298 K, except for CMC studies that were performed at a variety of concentrations. Quantitative ^1^H NMR data were obtained for a D_2_O/5.0% EtOH solutions of an SSA or 1 : 1 cisplatin co-formulation (5.5 mM) at 298 K. Here % values represent the proportion of compound to become NMR silent, and thus adopt solid like properties as this proportion of molecular species are incorporated into larger self-associated aggregates. All quantitative ^1^H NMR experiments were conducted with a delay time (*d*_1_) of 60 s. Average *D*_H_ measurements were obtained from DLS intensity particle size distribution peak maxima

Compound	Quantitative ^1^H NMR (%)		Zeta potential (mV)	*D*_H_ (nm)	CMC (mM)
	SSA anion	SSA cation			
1 only	51 (ref. 7)	50 (ref. 7)	−67 (ref. 10)	142 (ref. 7)	10.4 (ref. 7)
1 + cisplatin	65 (ref. 10)	83 (ref. 10)	−42 (ref. 10)	161 (ref. 10)	3.3 (ref. 10)
6 only	10 (ref. 7)	8 (ref. 7)	−44 (ref. 10)	300 (ref. 7)	0.5 (ref. 7)
6 + cisplatin	63	85	−53	240	2.0

Following this physiochemical investigation, the synergistic cytotoxic effects of these SSA/cisplatin combinations were determined using the Chou-Talalay method.^18^ Chou-Talalay analysis yields a combination index (CI) where, for this study; if CI > 1.05 the drug combination is antagonistic; if CI = 0.95–1.05 the effects of the drug combination is additive, and where values of CI < 0.95 are indicative of synergy. Building on our results from previous SSA combination therapy investigations, the SSA to be investigated was preincubated with the A2780 cells for one hour before the cisplatin was introduced to the cell culture. This procedure has previously been shown to be the most effective in inducing synergy between the SSA and a second therapeutic agent, such as cisplatin.^10^

**Fig. 4:**
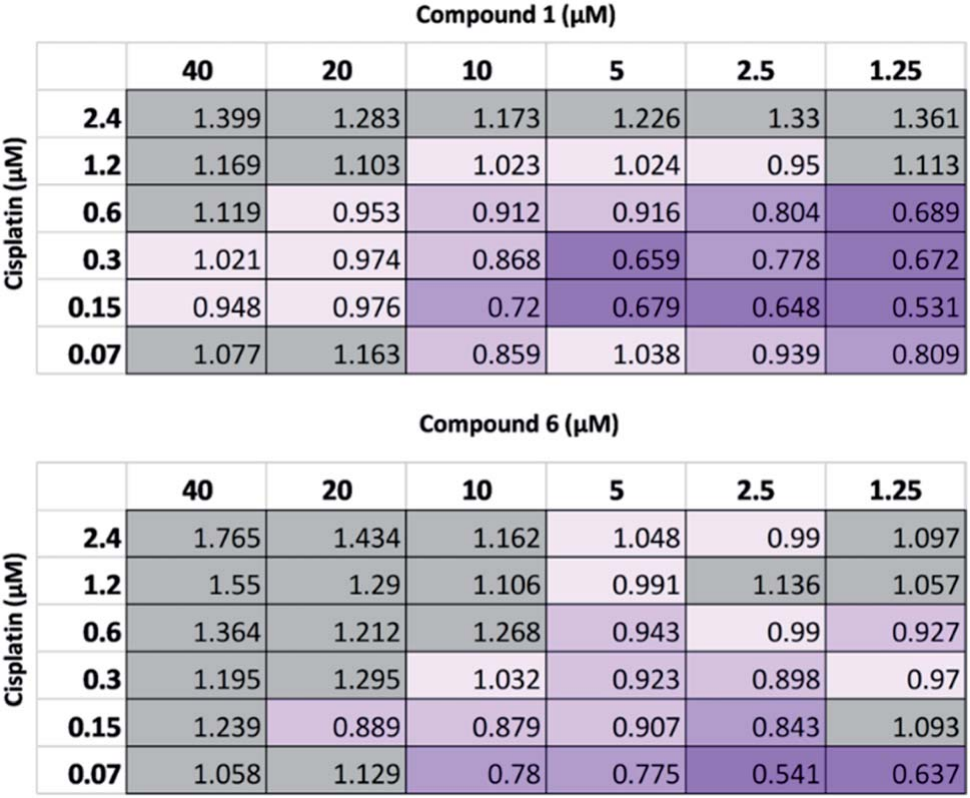
CI values obtained for 1 and 6 pre-incubated with A2780 cells for one hour before addition of cisplatin and then 96 hour incubation, followed by SRB assay. Each assay comprised *n* = 3 technical replicates and is representative of three independent experiments. 0.30–0.70 = synergism; 0.70–0.85 = moderate synergism; 0.85–0.95 = slight synergism; 0.95–1.05 = additive; >1.05 = antagonism.

As summarized in Fig. 4, at the highest concentrations of cisplatin or SSA used, these combinations yielded antagonistic or additive anticancer effects. Interestingly, at lower concentrations of both cisplatin and SSA, synergism was observed. The maximum synergy for cisplatin and 1 was observed at 0.15 μM and 1.25 μM respectively, yielding CI = 0.531. Similar synergism was displayed between cisplatin and 6; CI = 0.541 at concentrations of 0.07 μM of cisplatin and 2.5 μM of 6. However, a limitation of this method is that at the lowest concentration of either drug used, both must still exhibit a cytotoxic cell effect. As such, a modified assay was performed, using a lower concentration range of 1 whilst maintaining the concentration of cisplatin. Whilst 2 μM and 4 μM of 1 had minimal effects on cell growth alone (Fig. S26†), they enhanced the activity of cisplatin, which correlates with the synergistic effects shown in Fig. 4 for combining 2.5 μM and 5 μM of 1 with cisplatin. In contrast, 0.2 μM and 0.8 μM of 1 antagonised the growth inhibitory effects of cisplatin. This indicates that the synergistic activity of 1 with cisplatin is dose-dependent.

To ensure the suitability of these SSAs (1–7) as potential drug efficacy enhancers, preliminary toxicity studies were also conducted against normal human dermal fibroblasts. Using both confluency and cell viability assays it was determined that the inherent cytotoxicity of these compounds would not inhibit SSA use towards these applications, as all SSAs displayed no significant effect in these assays, even at concentrations 10-fold higher than those concentrations at which synergy was observed (Fig. S27 and S28†).

Finally, to gain greater insight into the synergistic mode of action of SSAs, western blot analysis was performed on lysates prepared from A2780 cells treated with either 1 or cisplatin alone at high (10 × GI_50_) and low (1 × GI_50_) concentrations and in combination. Several cellular proteins were analysed to evaluate effects on key intracellular signalling pathways. These were; cleaved-PARP (PARP C), a cellular indicator of apoptosis,^19^ the cell signalling kinases ERK and AKT, and γH2AX, a biomarker of DNA damage.^20^

**Fig. 5:**
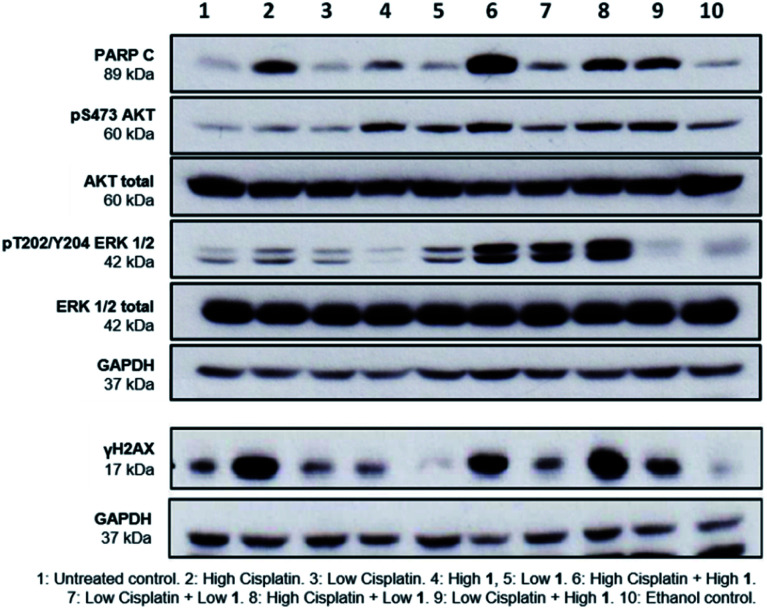
Western blot of A2780 cells treated with high (approx. 10 × GI_50_) and low (GI_50_) concentrations of 1 and cisplatin, after 24 hours incubation. GAPDH used as loading control. Data representative of *n* = 3.

PARP C production in response to 1 alone is comparable to controls, suggesting that at the concentrations shown, 1 does not induce apoptosis. Cisplatin alone induced apoptosis (Fig. 5) but interestingly, the combination of 1 and cisplatin shows an increase in PARP C levels compared to that of cisplatin alone, supporting the hypothesis that the SSA compounds may be used to enhance cancer drug efficacy. This decrease of effective dose is crucial towards reducing the systemic toxicity inherent in many cancer therapies. Compound 1 alone (but not cisplatin) induced phosphorylation of AKT, an indicator of AKT activation, but this was not further enhanced when 1 and cisplatin were combined. Both compounds had limited effects on phosphorylated ERK, an indicator of ERK activity but there was a strong induction of ERK activation when 1 and cisplatin were combined. Strong ERK activation has been reported to correlate with apoptosis.^21^ Interestingly, the combination of low cisplatin with high 1 showed a higher γH2AX signal compared to low cisplatin alone, indicative of a synergistic effect of 1 on cisplatin mediated DNA damage.

## Conclusions

In summary, reducing the effective dose of commonly used anticancer agents is a crucial challenge in medicine, to diminish the potentially life-threatening side effects that are a result of current dosing strategies. SSAs 1 and 6 have shown promise as agents that increase the efficacy of cisplatin and offer a potential platform for the targeted delivery of other therapeutics. These investigations remain ongoing within our research groups.

## Author contributions

Nova O. Dora: investigation; validation. Edith Blackburn: investigation; supervision; validation. Jessica E. Boles: investigation; validation. George T. Williams: funding acquisition; validation; roles/writing – original draft. Lisa J. White: investigation; validation. Scarlett E. G. Turner: investigation; validation. J. Daniel Hothersall: investigation; validation. Trevor Askwith: methodology; project administration; supervision. Jack A. Doolan: roles/writing – original draft. Daniel P. Mulvhill: investigation; resources; validation. Michelle D. Garrett: conceptualization; funding acquisition; methodology; project administration; supervision; writing – review & editing. Jennifer R. Hiscock: conceptualization; funding acquisition; methodology; project administration; supervision; writing – review & editing.

## Conflicts of interest

There are no conflicts to declare.

## Supplementary Material

RA-011-D1RA02281D-s001
