# Supplementary material for: Supramolecular self-associating amphiphiles (SSAs) as nanoscale enhancers of cisplatin anticancer activity
Source: RSC Adv. 2021 Apr 15;11(23):14213–7. doi: 10.1039/d1ra02281d (PMC8697675; doi:10.1039/d1ra02281d)
Supplement: RA-011-D1RA02281D-s001 [file RA-011-D1RA02281D-s001.pdf]

Electronic Supplementary Material (ESI) for RSC Advances.
This journal is © The Royal Society of Chemistry 2021

Supramolecular self-associating amphiphiles (SSAs) as nanoscale enhancers of cisplatin anticancer activity

Nova O. Dora, Edith Blackburn, Jessica E. Boles, George T. Williams, Lisa J. White, J. Daniel Hothersall, Trevor Askwith, Scarlett E. G. Turner, Jack A. Doolan, Daniel P. Mulvhill, Michelle D. Garrett* and Jennifer R. Hiscock*

Contents

Chemical Experimental 2
- General remarks: 2
- DLS Studies: 2
- Zeta Potential Studies 2
- Tensiometry Studies: 2
- Quantitative ^{1}H NMR: 2

Biological Experimental 2
- Cell culture: 2
- SRB Assay: 2
- Drug combination studies: 3
- Studies with Human dermal fibroblasts: 3
- Immunofluorescence: 3
- Western Blot Analysis: 4

Chemical Synthesis 9
NMR – Structural confirmation 10
^{1}H NMR Quantitative Experiments 17
Stability data 19
Dynamic light scattering data 21
Surface Tension and Stability Data 23
Drug Combination Assay 25
Cell confluency assay 26
Cell viability assay 26
References 27

Chemical Experimental

General remarks: A positive pressure of nitrogen and oven dried glassware were used for all reactions. All solvents and starting materials were purchased from known chemical suppliers or available stores and used without any further purification unless specifically stipulated. The NMR spectra were obtained using a Burker AV2 400 MHz or AVNEO 400 MHz spectrometer. The data was processed using ACD Labs, MestReNova or Topspin software. NMR Chemical shift values are reported in parts per million (ppm) and calibrated to the centre of the residual solvent peak set (s = singlet, br = broad, d = doublet, t = triplet, q = quartet, m = multiplet). Tensiometry measurements were undertaken using the Biolin Scientific Theta Attension optical tensiometer. The data was processed using Biolin OneAttension software. A Hamilton (309) syringe was used for the measurements. DLS and Zeta Potential studies were carried out using Anton Paar Litesizer^{TM} 500 and processed using KalliopeTM Professional. Cellular growth curve measurements obtained using Thermo Scientific Multiscan Go 1510-0318C plate reader and recorded using the SkanIt Software 4.0 and a Clariostar plater reader using MARS data analysis software.

DLS Studies: All vials used for preparing the samples were clean and dry. All solvents used were filtered to remove any particulates that may interfere with the results obtained. Samples of differing concentrations were obtained through serial dilution of a concentrated solution. All samples underwent an annealing process, in which they were heated to 40 °C before being allowed to cool to 25 °C. A series of 9 or 10 runs were recorded at 25 °C.

Zeta Potential Studies: All vials used for preparing the samples were clean and dry. All solvents used were filtered to remove any particulates that may interfere with the results obtained. All samples underwent an annealing process in which the various solutions were heated to approximately 40 °C before cooling to room temperature, allowing each sample to reach a thermodynamic minimum. The final zeta potential value given is an average of the number of experiments conducted at 25 °C.

Tensiometry Studies: All the samples were prepared in an EtOH: H_2O (1:19) solution. All samples underwent an annealing process in which the various solutions were heated to approximately 40 °C before being allowed to cool to room temperature, allowing each sample to reach a thermodynamic minimum. All samples were prepared through serial dilution of the most concentrated sample. Three surface tension measurements were obtained for each sample at a given concentration, using the pendant drop method. The average values were then used to calculate the critical micelle concentration (CMC).

Quantitative ^{1}H NMR: A ^{1}H NMR spectrum was obtained with a delay (d_1 = 60 s) for an SSA (5.5 mM) or 1:1 co-formulation of an SSA (5.5 mM) with cisplatin (5.5 mM) in D_2O/ 5.0 % EtOH. Through comparative integration of the SSA anionic and cationic component signals with the ethanol signal, the proportion of these SSA components to become 'lost' from solution, through the adoption of solid-like characteristics can be calculated.

Biological Experimental

Cell culture: The A2780 human ovarian carcinoma and U87MG human glioblastoma human cell lines were previously purchased from the Health Protection Agency (Salisbury, UK) and American Type Culture Collection (ATCC, USA) respectively. Both cell lines were cultured in Iscove's Modified Dulbecco's Medium (IMDM) supplemented with 10% Fetal bovine serum (FBS) at 37 °C and 5% CO_2.

SRB Assay: Cells were seeded into 96-well plates at 800 cells per well [(cpw), A2780 cell line] or 6,400 cpw (U87MG cell line) in cell culture medium and cultured for 48 hours followed by addition of each compound

over an eight point concentration range (each concentration in triplicate). Plates were then cultured for a further 96 h after which cell culture medium was removed from each well and cells fixed with addition of 70 μL/well of 10% (w/v) trichloroacetic acid (TCA) in distilled water followed by 30 mins incubation at room temperature. Each plate was then washed with distilled water five times before addition of 70 μL SRB dye (0.4% (w/v) sulforhodamine B (SRB) dye (ThermoFisher Scientific, USA) solubilised in 1% (v/v) acetic acid/distilled water) and incubation for 30 mins at room temperature, followed by washing three times with 1% (v/v) acetic acid and drying in a 37 °C oven overnight. Once dry, 100 μl of 10 mM Tris-base (ThermoFisher Scientific, USA) was added to each well and plates put on a microplate shaker for 10 mins at 200 rpm to solubilize the dye. Absorbance values were then read at wavelength 490 nm on a Victor X4 multi-label plate reader (PerkinElmer Life Sciences, USA), data analysed using Microsoft Excel and graphs produced using GraphPad Prism 6.0 and the growth inhibitory IC_{50} (GI_{50}) value calculated. The GI_{50} is defined as the compound concentration required to reduce cell growth by 50% versus untreated cells.

Drug combination studies: The dose effect relationship of cisplatin in combination with compound **1** or **6** was initially studied using the Chou-Talalay method.[5] A2780 cells were seeded in 96-well plates at 800 cpw and cultured for 48 hours after which 1.25 – 40 μM of either **1** or **6** was added for 1 hour, followed by addition of 0.075– 2.4 μM **cisplatin** to form a compound concentration matrix of cisplatin versus **1** or **6**. Cells were cultured for a further 96 hours then fixed, stained, absorbance read and data analysed as per SRB assay above.

Drug combinations were further explored at lower concentrations of **1**. A2780 cells were seeded at 800 cpw in 96 well plates and cultured for 48 hours after which 0.2 -8 μM **1** was added for one hour, followed by addition of 0.075 – 2.4 μM cisplatin. Plates were then cultured for 96 hours before being fixed and stained and data analysed as per SRB assay above.

Studies with Human dermal fibroblasts: Normal human dermal fibroblasts (PromoCell GmbH) were routinely cultured in DMEM supplemented with 10 % foetal calf serum at 37 °C at an atmosphere of 5 % CO2. Cells were seeded on white clear bottomed 96 well plates at 5,000 cells per well (200 µL) and grown for 16-20 hours. Medium was then replaced with low serum DMEM (0.1 % FCS) and test compounds were added in duplicate (with a final vehicle concentration of 0.3 % DMSO). Cells were incubated for 48 hours with images taken every 2 hours using the IncuCyte S3 to quantify percentage confluency changes (using image analysis algorithms). Cell viability was then assayed using CellTitre-Glo reagent and luminescence read (no wavelength parameters with luminescence module used) using a Pherestar plate reader. Staurosporine (500 nM) was used as a positive control to demonstrate cytotoxicity. For more detail regarding human dermal fibroblast experimental protocol please contact e-mail address given.‡

Immunofluorescence: A2780 cells were plated at 0.75 x 10^6 cells and U87MG cells at 0.6 x 10^6 cells per 35 mm imaging dish (µ-Dish 35 mm, Thistle Scientific, UK) for live cell imaging and cultured for 24 hours. Either **6** or **7** were added to each well at 90 μM or 180 μM respectively and cells examined immediately and then after one-hour exposure to compound. Samples were visualised using an Olympus IX71 microscope with PlanApo 100x OTIRFM-SP 1.45 NA lens mounted on a PIFOC z-axis focus drive (Physik Instrumente, Karlsruhe, Germany), and illuminated using LED light sources (Cairn Research Ltd, Faversham, UK) with appropriate filters (Chroma, Bellows Falls, VT). An Optosplit device (Cairn Research Ltd) was used to allow simultaneous acquisition of signals from two fluorophores that emitted light of different wavelengths. Samples were visualised using a QuantEM (Photometrics) EMCCD camera, and the system was controlled with Metamorph software (Molecular Devices). Each 3D-maximum projection of volume data was calculated from 21 z-plane images, each 0.2 µm apart, and analysed using Metamorph software.

Western Blot Analysis: A2780 cells were plated at $1x10^6$ cells in 10 cm dishes and cultured for 48 h followed by addition of 1 or 10 μM cisplatin or 1 or 10 μM of **1** alone or in combination with each other. Cells were cultured for a further 24 hours then washed with ice cold PBS, followed by addition of 100 μL lysis buffer (50 mM HEPES pH 7.4, 250 mM NaCl, 0.1% (v/v) Nonidet-P40, 1 mM DTT, 1 mM EDTA, 1 mM NAF, 10 mM β-glycerophosphate, 0.1 mM sodium orthovanadate, and Complete™ protease inhibitor cocktail (Roche, Switzerland) cells scraped off the plate, collected in a 1.5 ml microcentrifuge tube and incubated on ice for 30 minutes. Lysates were then centrifuged at 14,000 x g for 10 mins at 4°C, to clear any insoluble material, and cleared lysate transferred to a clean microcentrifuge tube, which was either kept on ice for immediate use or snap-frozen on dry ice and stored at -80°C for future use. Sample protein concentration was determined using the Bradford assay.[5] Samples were then normalized to an equal protein concentration using lysis buffer and 5x sample buffer (0.3M Tris pH6.8, 50% [v/v] glycerol [ThermoFisher Scientific, UK] 25% [v/v] β-mercaptoethanol, 10% [w/v] SDS [ThermoFisher Scientific, UK] and 0.05% [v/v] bromophenol blue) added to each sample.

The samples were then mixed and heated at 95 °C for 5 minutes before being electrophoresed on 12% tris-glycine (resolving) gels at 150 V for approximately 1 h in Running Buffer (25 mM Tris-HCl, 192 mM Glycine, 0.1% (w/v) SDS). Proteins were then transferred to a polyvinylidene difluoride (PVDF) membrane through wet transfer at 100 V for 90 mins in Transfer Buffer (25 mM Tris-base, 192 mM glycine, 10% [v/v] methanol). After protein transfer, the PVDF membrane was blocked in Blocking Buffer containing 5% (w/v) powdered milk in TBST (50 mM Tris pH 8.0, 150 mM NaCl, 0.1% [v/v] Tween-20) for 1 hour and then incubated in primary antibody (see **Table S1** for list of antibodies used) in Blocking Buffer overnight at 4 °C (Gamma H2AX antibody incubated in 5% (w/v) BSA in TBST). Membranes were then washed 4 x 5 minutes with TBST, incubated with HRP-conjugated secondary antibody in Blocking Buffer for 1 hour at room temperature. Membranes were then washed 4 x 5 minutes in TBST before being treated with Clarity™ Western ECL Blotting Substrate (Bio-Rad, USA) as per manufacturer's instructions and then exposed to Amersham Hyperfilm (GE Healthcare, UK) and processed using an Optimax™ 2010 film processor (Protec, Germany) in a dark room.

Table S1 - List of antibodies used in the western blot analysis.

**Primary antibody**	**Supplier**	**Catalogue number**	**Species**	**Dilution**
AKT	Cell signalling	4691	Rabbit	1:5,000
AKT pSer473	Cell signalling	4060	Rabbit	1:1,000
ERK 1/2	Cell signalling	4695	Rabbit	1:1,000
ERK1/2 pT202/Y204	Cell signalling	4370	Rabbit	1:2,000
PARP C	Cell signalling	9541	Rabbit	1:1,000
Gamma H2AX	Cell signalling	05-636	Mouse	1:500
GAPDH	Millipore	MAB374	Mouse	1:100,000
**Secondary antibody**	**Supplier**	**Catalogue number**	**Species**	**Dilution**
Anti-mouse HRP conjugate	Bio-Rad	170-6516	Goat	1:10,000
Anti-rabbit HRP conjugate	Bio-Rad	170-6515	Goat	1:10,000

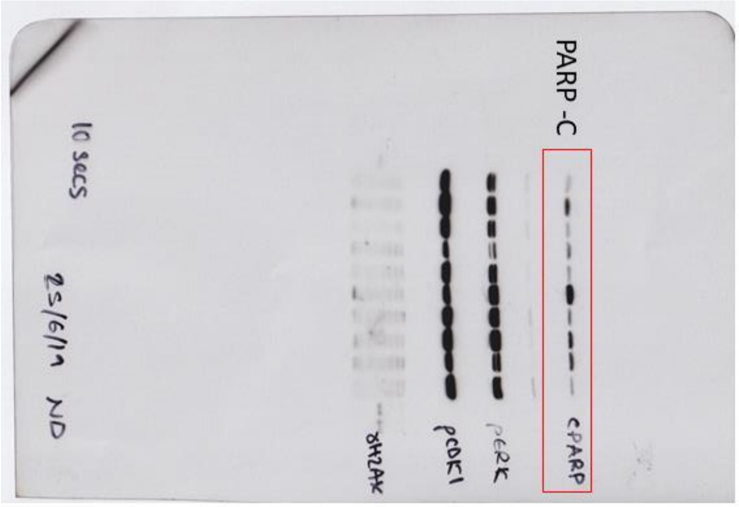

Figure S1 – Uncropped western blot. This image contains experimental data used within Figure 5, which has been outlined in RED.

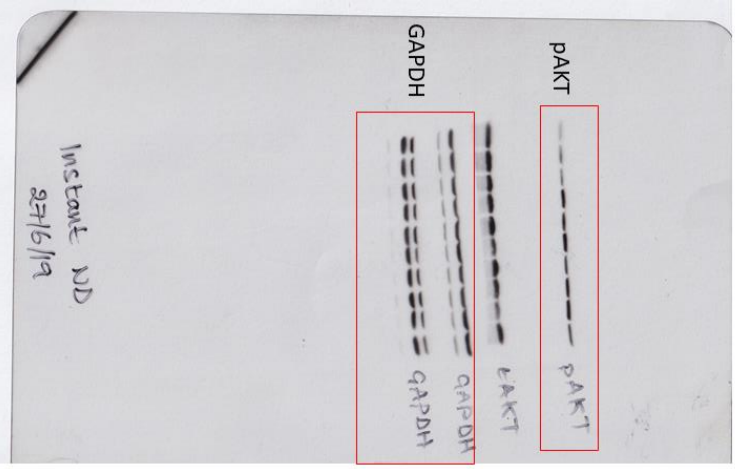

Figure S2 – Uncropped western blot. This image contains experimental data used within Figure 5, which has been outlined in RED.

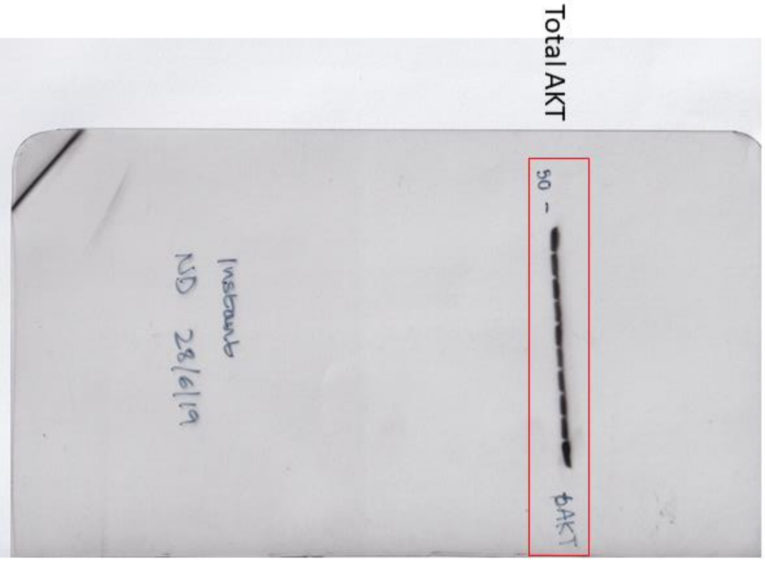

Figure S3 – Uncropped western blot. This image contains experimental data used within Figure 5, which has been outlined in RED.

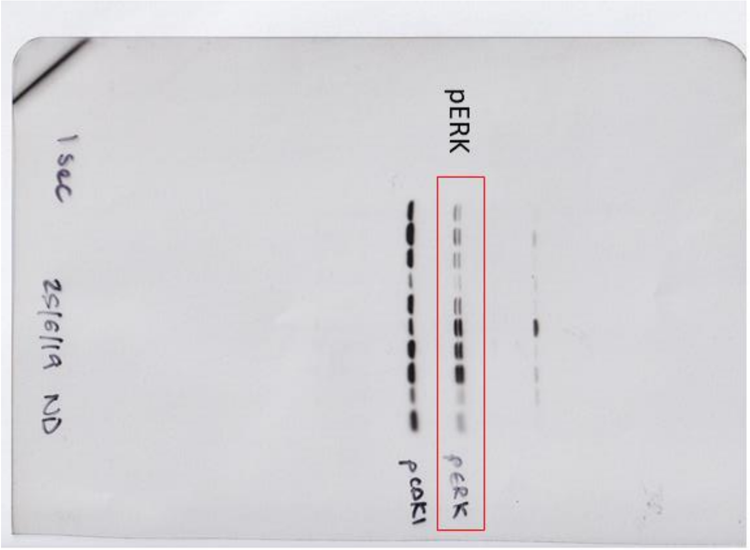

Figure S4 – Uncropped western blot. This image contains experimental data used within Figure 5, which has been outlined in RED.

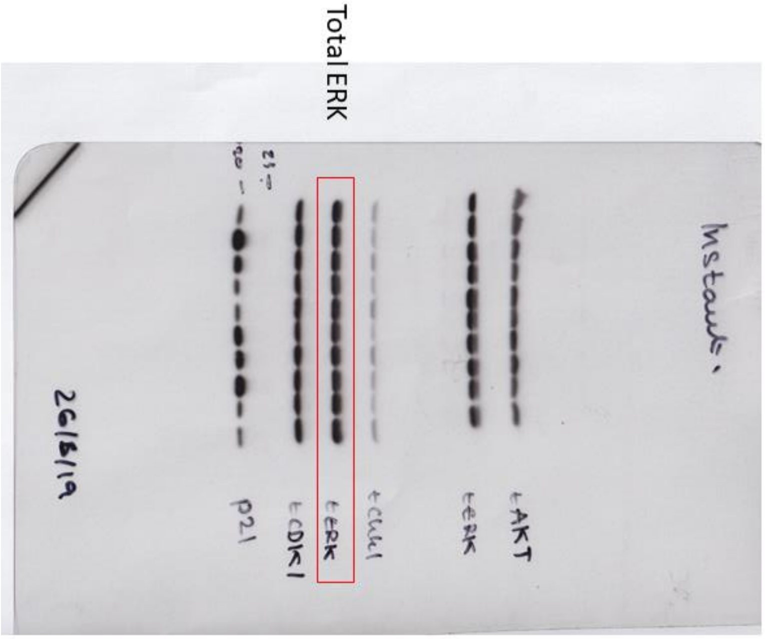

Figure S5 – Uncropped western blot. This image contains experimental data used within Figure 5, which has been outlined in RED.

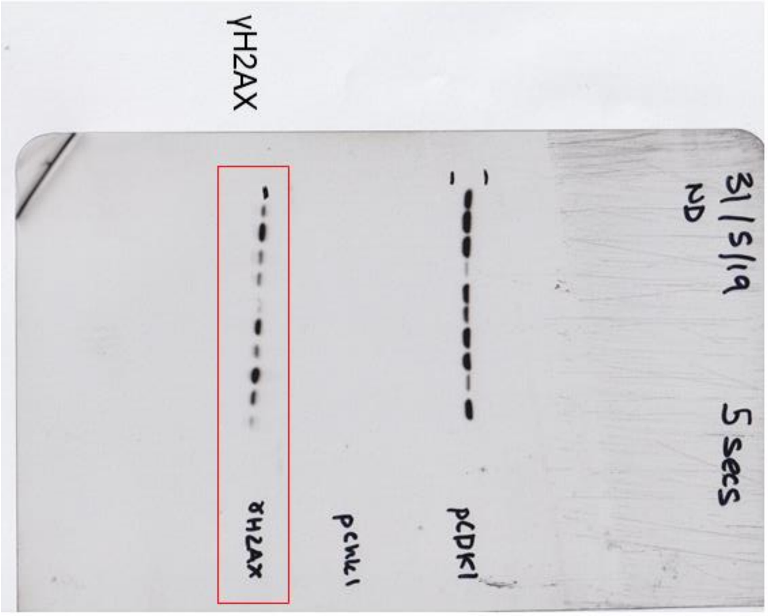

Figure S6 – Uncropped western blot. This image contains experimental data used within Figure 5, which has been outlined in RED.

Chemical Synthesis

Cisplatin was purchased commercially and used without further purification.

Compound **1**: This compound was synthesised in line with our previously published methods. Proton NMR were found to match our previously published values.[1] ^{1}H NMR (400 MHz, 298.15 K, DMSO-d_6): δ: 9.19 (s, 1H), 7.55 (s, 4H), 6.65 (s, 1H), 3.88 (d, *J* = 5.84 Hz, 2H), 3.18 - 3.13 (m, 8H), 1.60 - 1.56 (m, 8H), 1.31 - 1.29 (m, 8H), 0.93 (t, *J* = 7.32 Hz, 12H).

Compound **2**: This compound was synthesised in line with our previously published methods. Proton NMR were found to match our previously published values. [2] ^{1}H NMR (400 MHz, 298.15 K, DMSO-d_6): δ: 9.17 (s, 1H), 7.56 (s, 4H), 6.56 (s, 1H), 3.87 (d, *J* = 5.68 Hz, 2H), 3.09 (s, 12H).

Compound **3**: This compound was synthesised in line with our previously published methods. Proton NMR were found to match our previously published values. [2] ^{1}H NMR (400 MHz, 298.15 K, DMSO-d_6): δ: 9.19 (s, 1H), 7.55 (s, 4H), 6.68 (s, 1H), 3.89 (d, *J* = 5.39 Hz, 2H), 3.14 – 3.09 (m, 8H), 1.61 (q, *J* = 14.96 Hz, 7.31 Hz, 8H), 0.89 (t, *J* = 7.24 Hz, 12H).

Compound **4**: This compound was synthesised in line with our previously published methods. Proton NMR were found to match our previously published values. [2] ^{1}H NMR (400 MHz, 298.15 K, DMSO-d_6): δ: 8.23 (s, 1H), 7.00 (d, *J* = 8.60 Hz, 2H), 6.51 (d, *J* = 8.60 Hz, 2H), 6.17 (t, *J* = 5.24 Hz, 1H), 4.63 (s, NH), 3.81 (d, *J* = 5.80 Hz, 2H), 3.17 - 3.13 (m, 8H), 1.60 - 1.54 (m, 8H), 1.31 (m, 8H), 0.93 (t, *J* = 7.24 Hz, 12H).

Compound **5**: This compound was synthesised in line with our previously published methods. Proton NMR were found to match our previously published values. [2] ^{1}H NMR (400 MHz, 298.15 K, DMSO-d_6): δ: 9.4 (s, 1H), 7.60 (d, *J* = 8.52 Hz, 2H), 7.53 (d, *J* = 8.84 Hz, 2H), 6.54 (t, *J* = 5.40 Hz, 1H), 3.39 (t, *J* = 5.60 Hz, 2H), 3.15 (m, 8H), 2.57 (t, *J* = 6.24 Hz, 2H), 1.56 (m, 8H), 1.31 - 1.30 (m, 8H), 0.94 (t, *J* = 7.44 Hz, 12H).

Compound **6**: This compound was synthesised in line with our previously published methods. Proton NMR were found to match our previously published values. [3] ^{1}H NMR (400 MHz, 298.15 K, DMSO-d_6): δ: 9.14 (s, 1H), 7.94 – 7.86 (m, 4H), 7.55 (d, *J* = 8.72 Hz, 2H), 7.32 – 7.30 (m, 1H), 6.56 (s, 1H), 3.88 (d, *J* = 4.8 Hz, 2H), 3.18 – 3.14 (m, 9H), 2.44 (s, 3H), 1.60 – 1.56 (m, 8H), 1.31 – 1.26 (m, 8H), 0.93 (t, *J* = 7.44 Hz, 12H).

Compound **7**: This compound was synthesised in line with our previously published methods. Proton NMR were found to match our previously published values. [3] ^{1}H NMR (400 MHz, 298.15 K, DMSO-d_6): δ: 10.63 (s, 1H), 8.43 (s, 1H), 8.27 (d, *J* = 7.79 Hz, 1H), 8.15 (d, *J* = 7.92 Hz, 1H), 7.85 (d, *J* = 7.88 Hz, 2H), 7.58 (t, *J* = 7.58 Hz, 1H), 7.48 (t, *J* = 11.69, 7.49 Hz, 2H), 7.10 (s, 1H), 3.96 (s, 2H), 3.18 – 3.13 (m, 8H), 1.56 (m, 8H), 1.31 – 1.29 (m, 8H), 0.93 (t, *J* = 7.34 Hz, 12H).

NMR – Structural confirmation

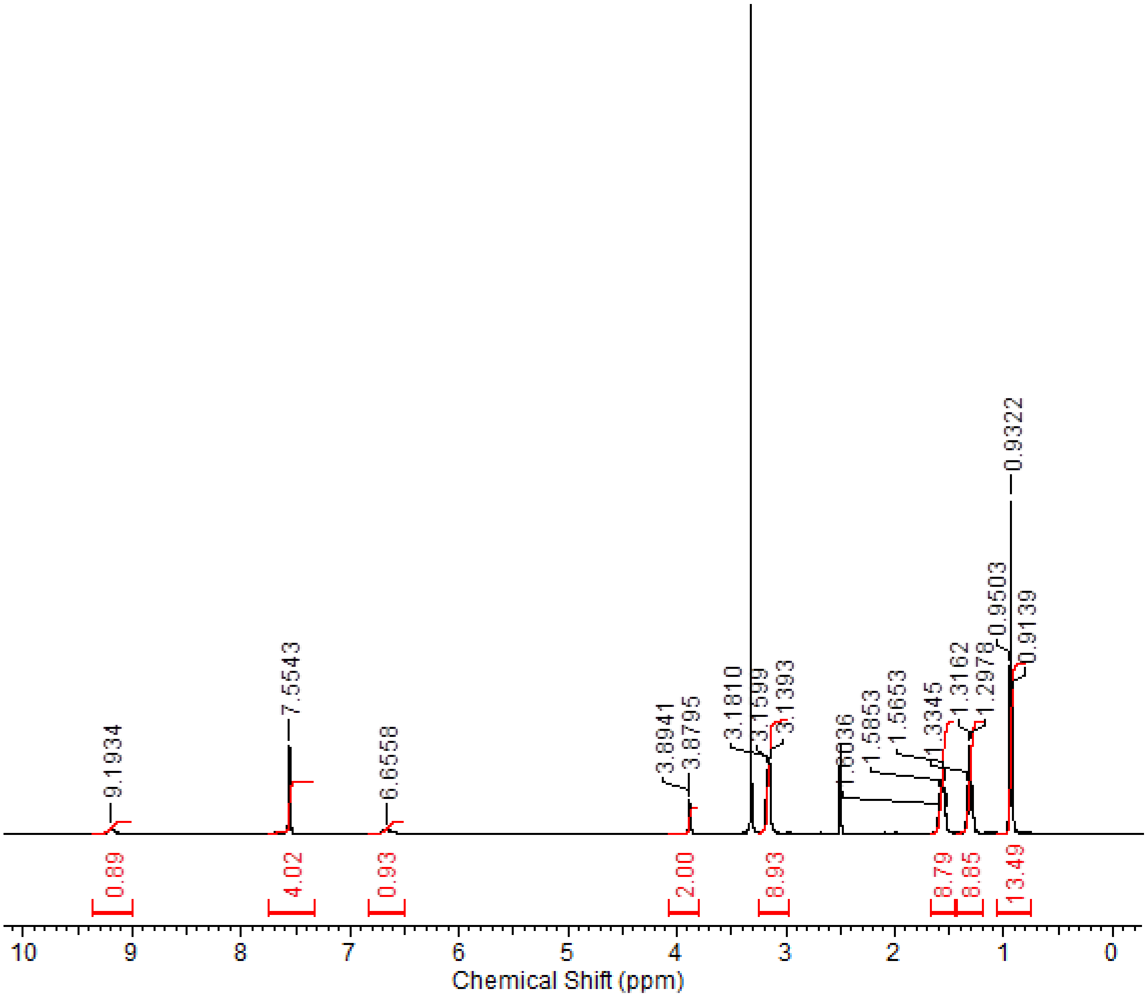

Figure S7 -^{1}H NMR of compound **1** in DMSO-d_6 conducted at 298.15 K.

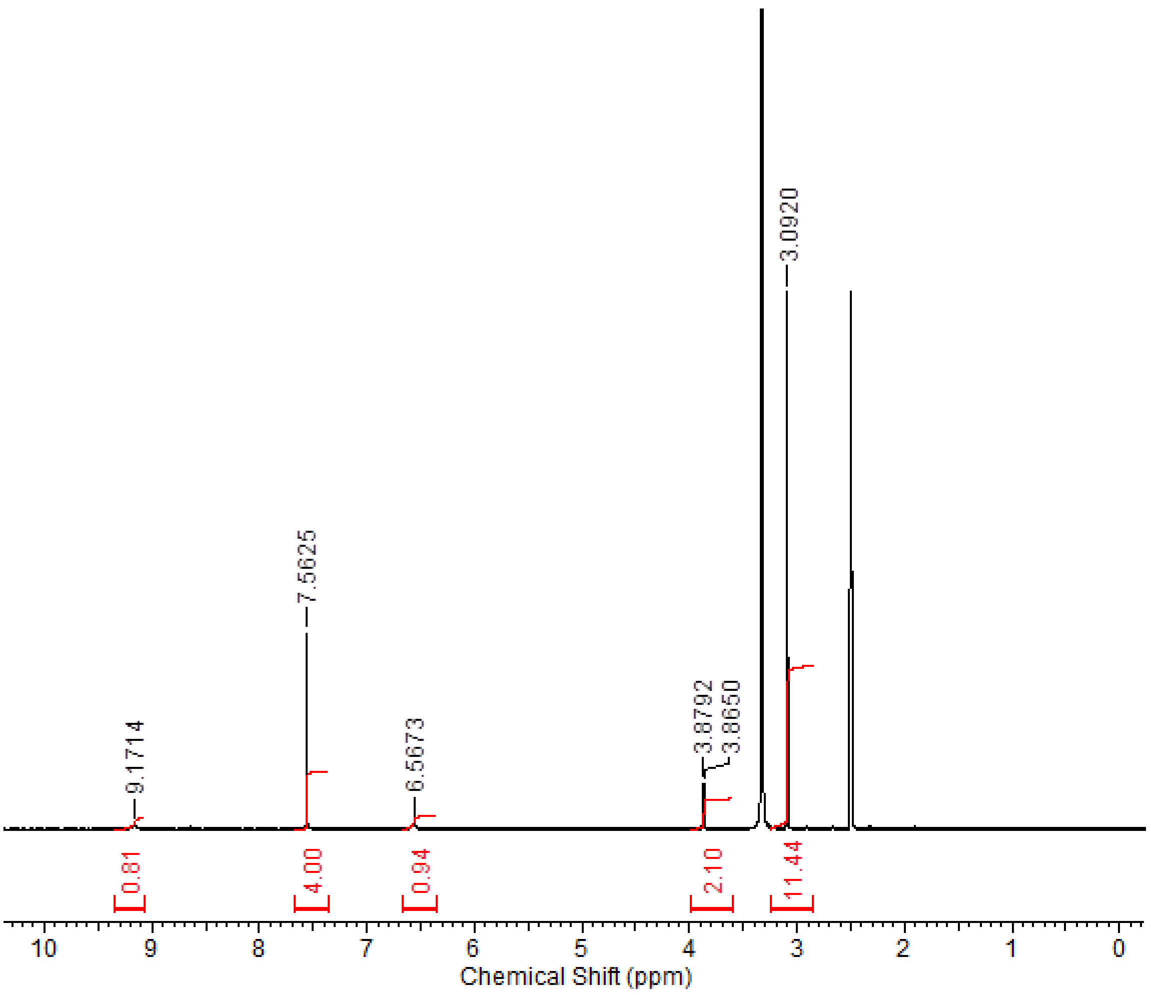

Figure S8 - ^{1}H NMR of compound **2** in DMSO-d_6 conducted at 298.15 K.

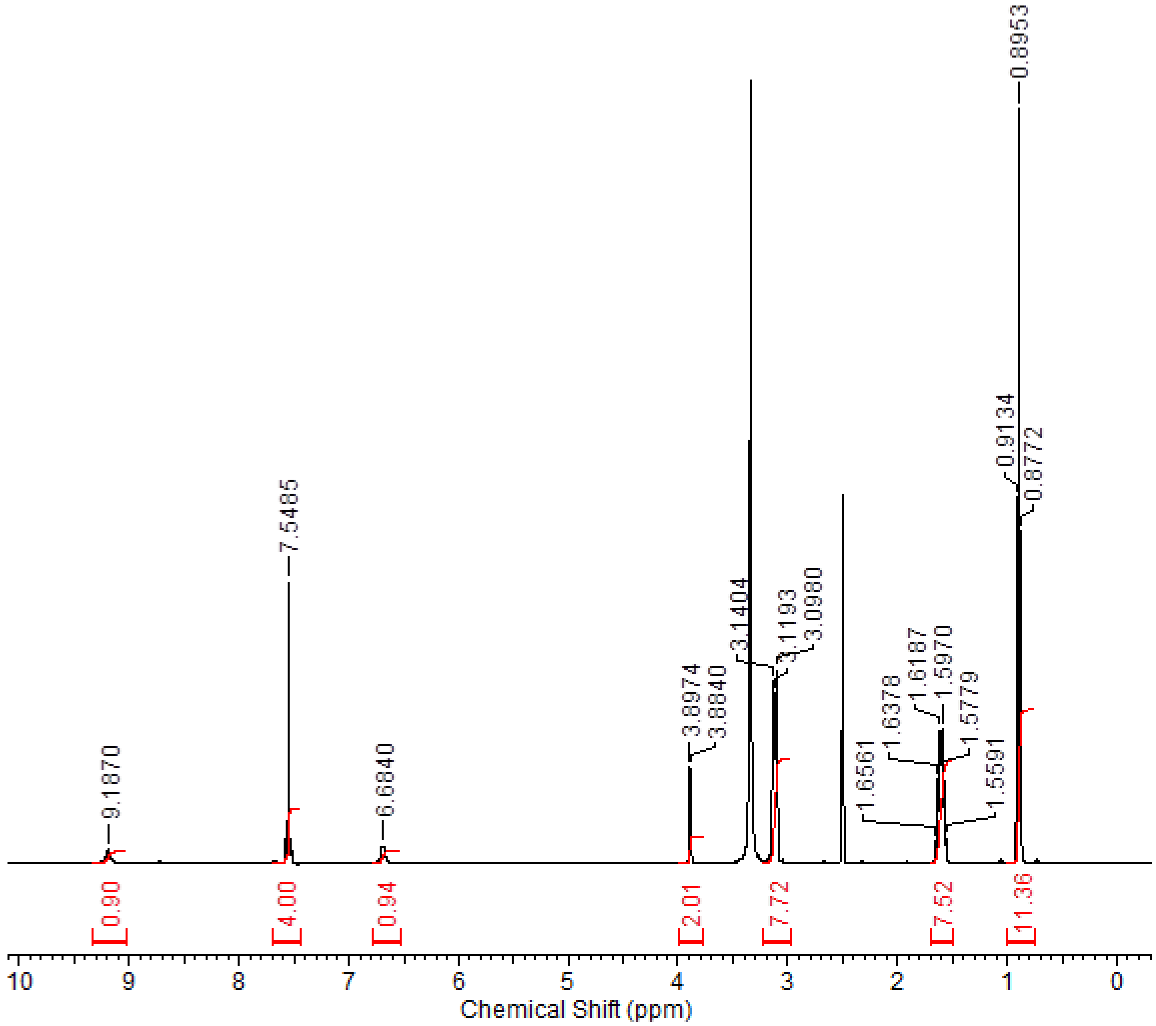

Figure S9 - ^{1}H NMR of compound **3** in DMSO-d_6 conducted at 298.15 K.

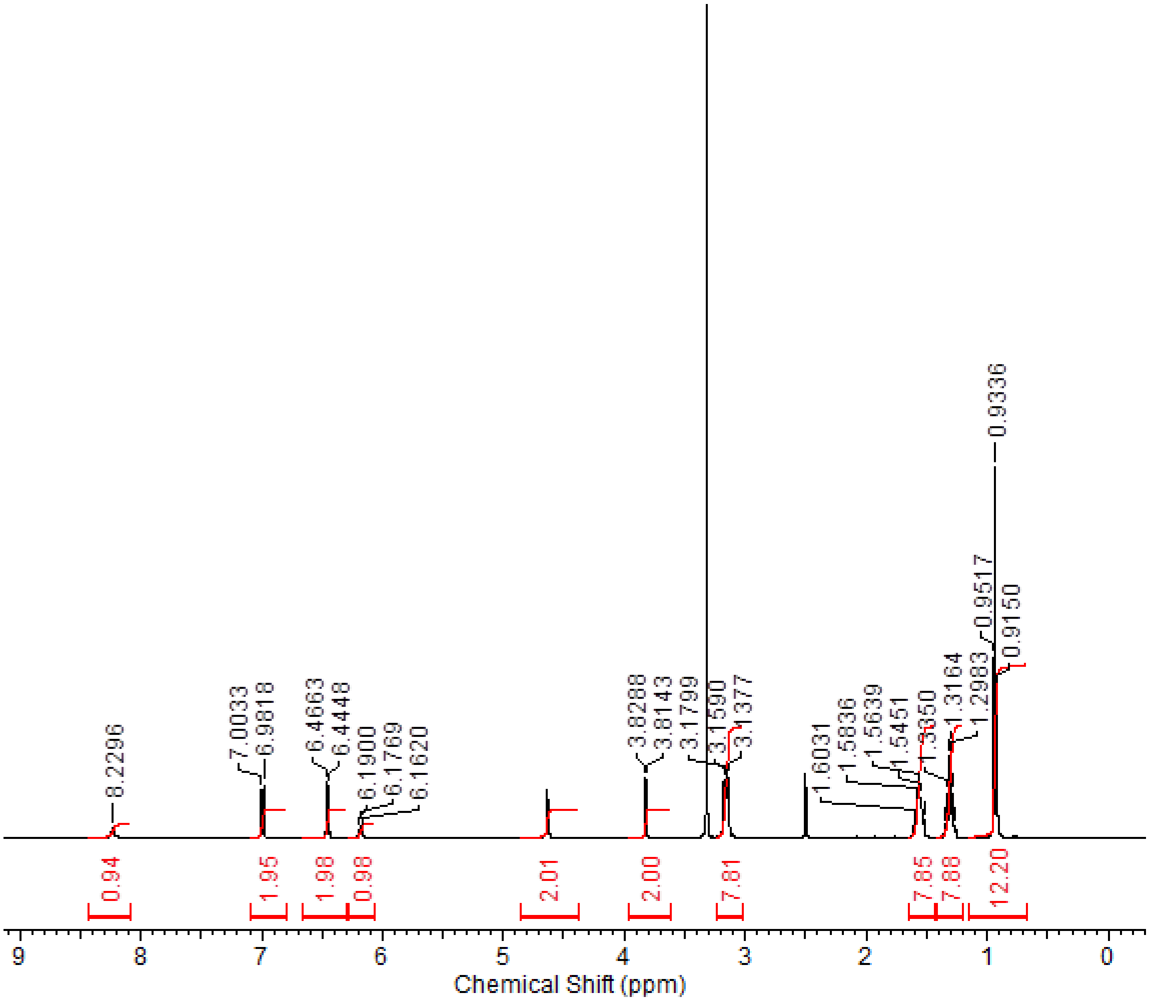

Figure S10 - ^{1}H NMR of compound **4** in DMSO-d_6 conducted at 298.15 K.

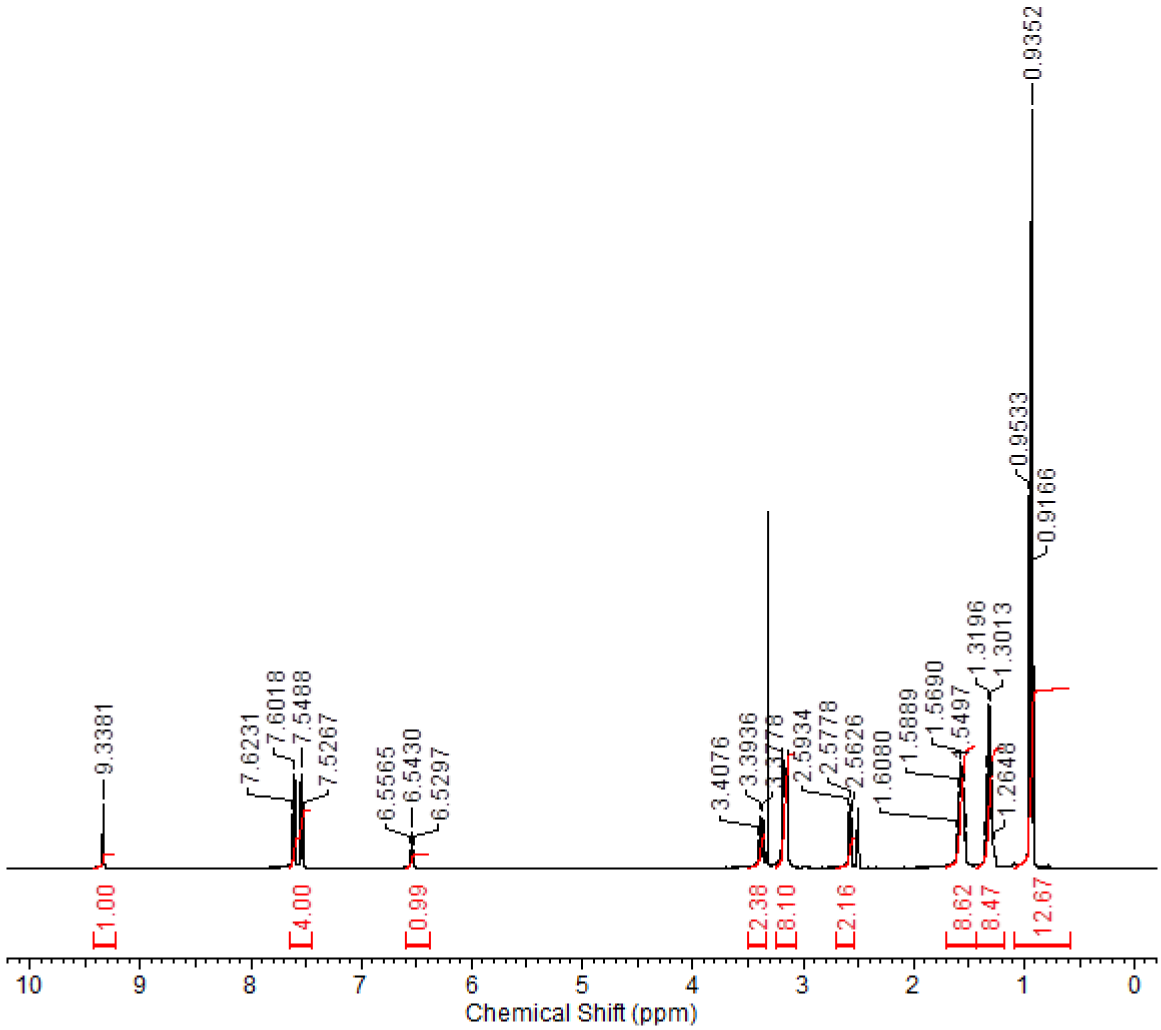

Figure S11 - ^{1}H NMR of compound **5** in DMSO-d_6 conducted at 298.15 K.

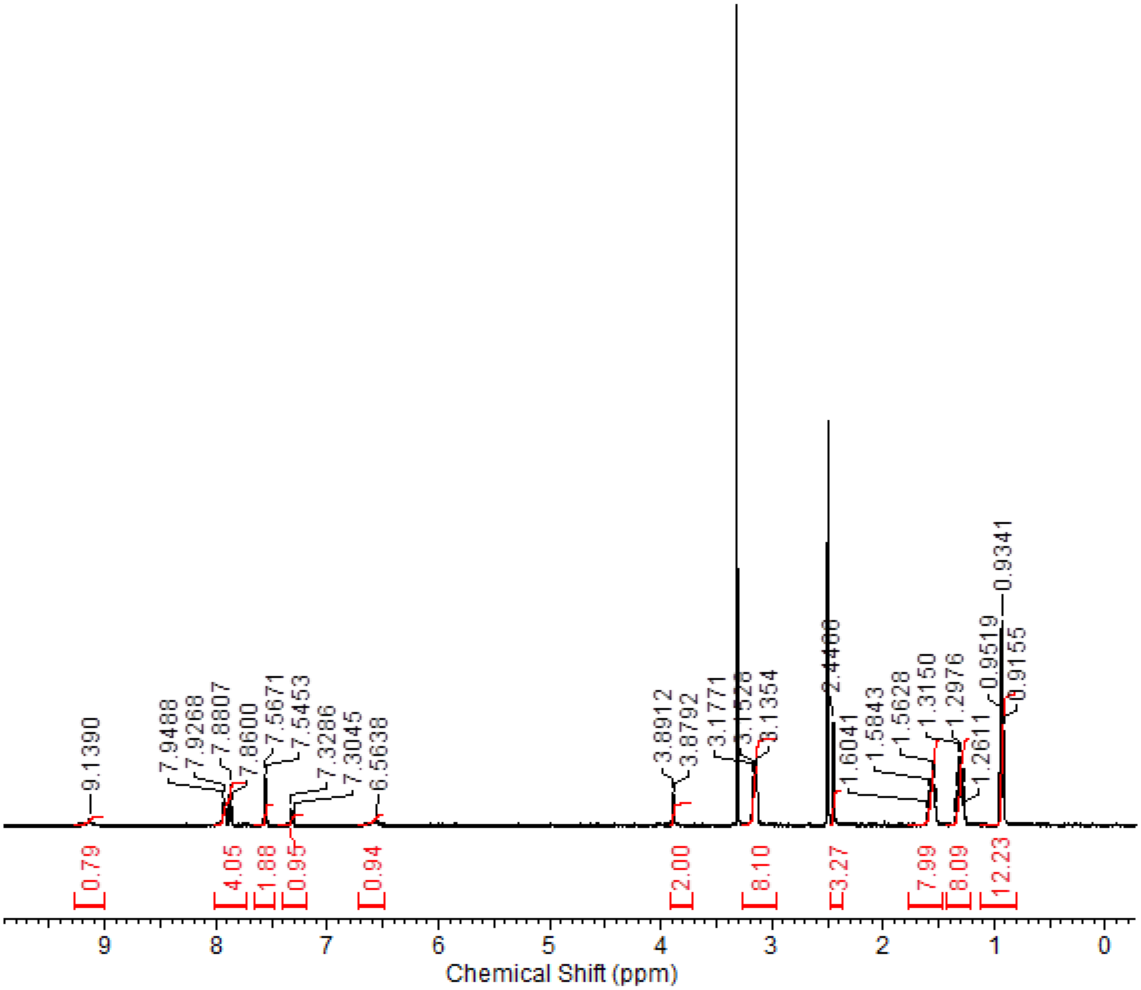

Figure S12 -^{1}H NMR of compound **6** in DMSO-d_6 conducted at 298.15 K.

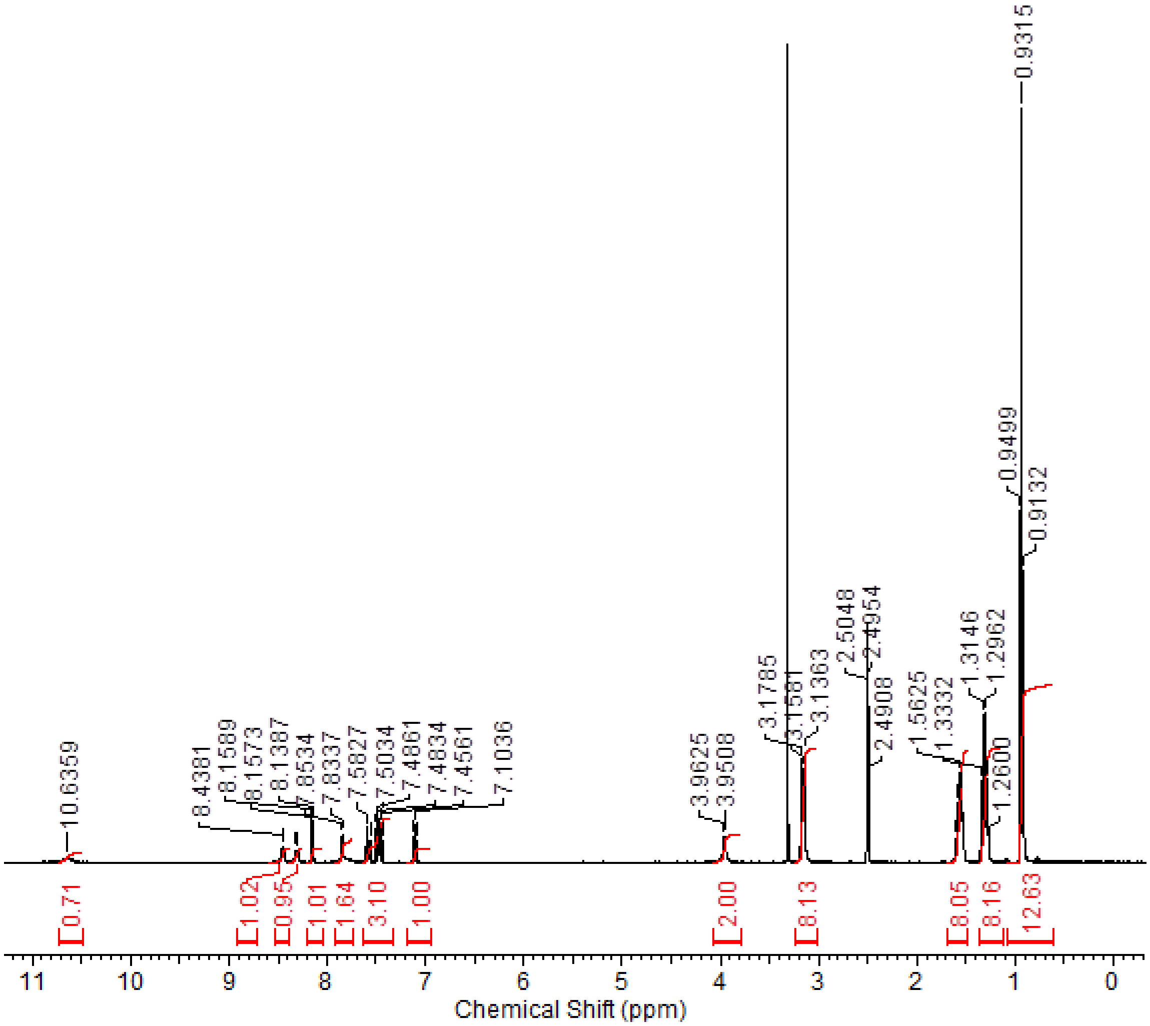

Figure S13 -^{1}H NMR of compound **7** in DMSO-d_6 conducted at 298.15 K.

^{1}H NMR Quantitative Experiments

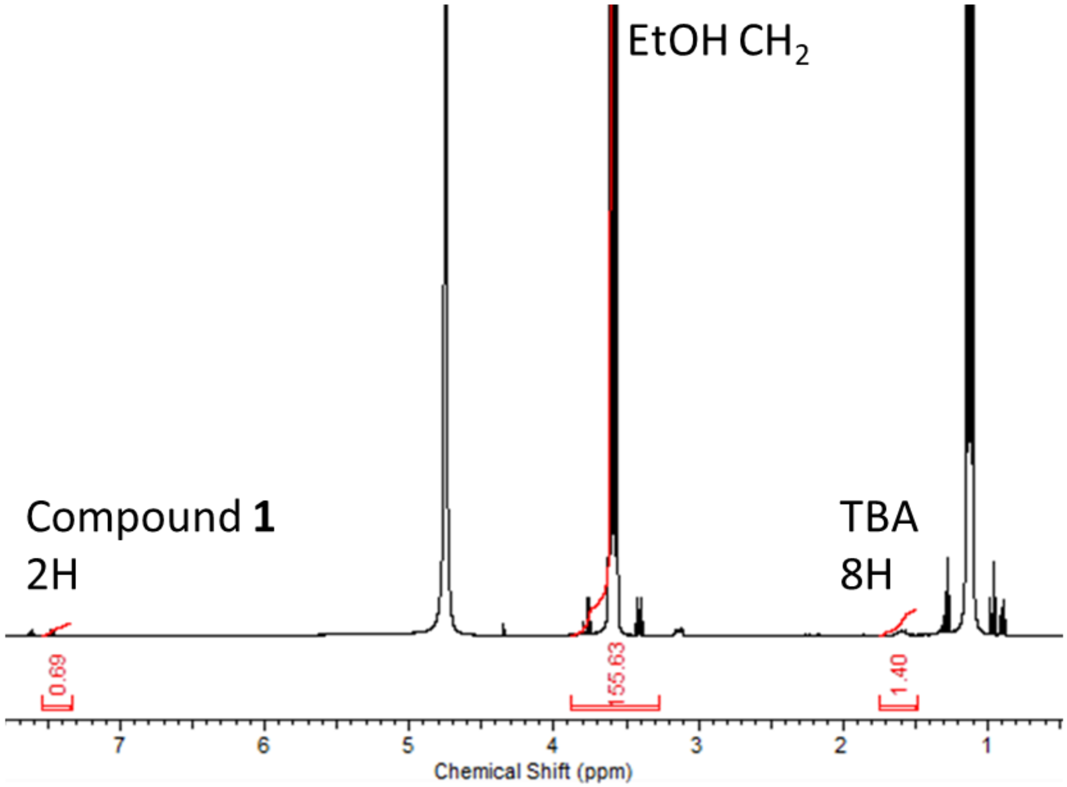

Figure S14 -^{1}H NMR spectrum with a delay (d_1 = 60 s) for a co-formulation of cisplatin and compound **1** (5.5 mM) in D_2O/ 5.0 % EtOH. An apparent 65 % loss of the anionic component and an 83 % loss of the cationic TBA component was observed upon comparative signal integration. Under the same experimental conditions, a 51 % loss of the anion and a 50 % loss of the cation was observed for compound **1** only.[2]

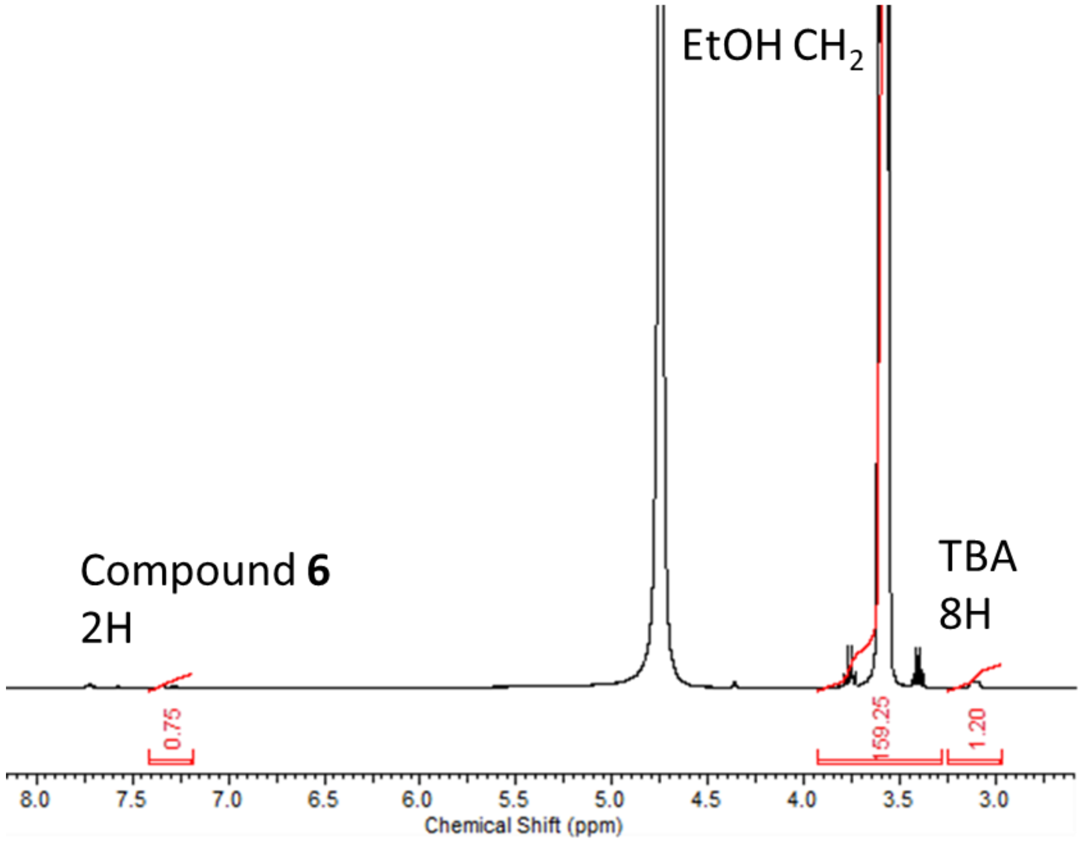

Figure S15 - ^{1}H NMR spectrum with a delay (d_1 = 60 s) for a co-formulation of cisplatin and compound **6** (5.5 mM) in D_2O/ 5.0 % EtOH. An apparent 63 % loss of the anionic component and an 85 % loss of the cationic TBA component was observed upon comparative signal integration. Under the same experimental conditions, a 10 % loss of the anion and an 8 % loss of the cation was observed for compound **6** only.[2]

Stability data

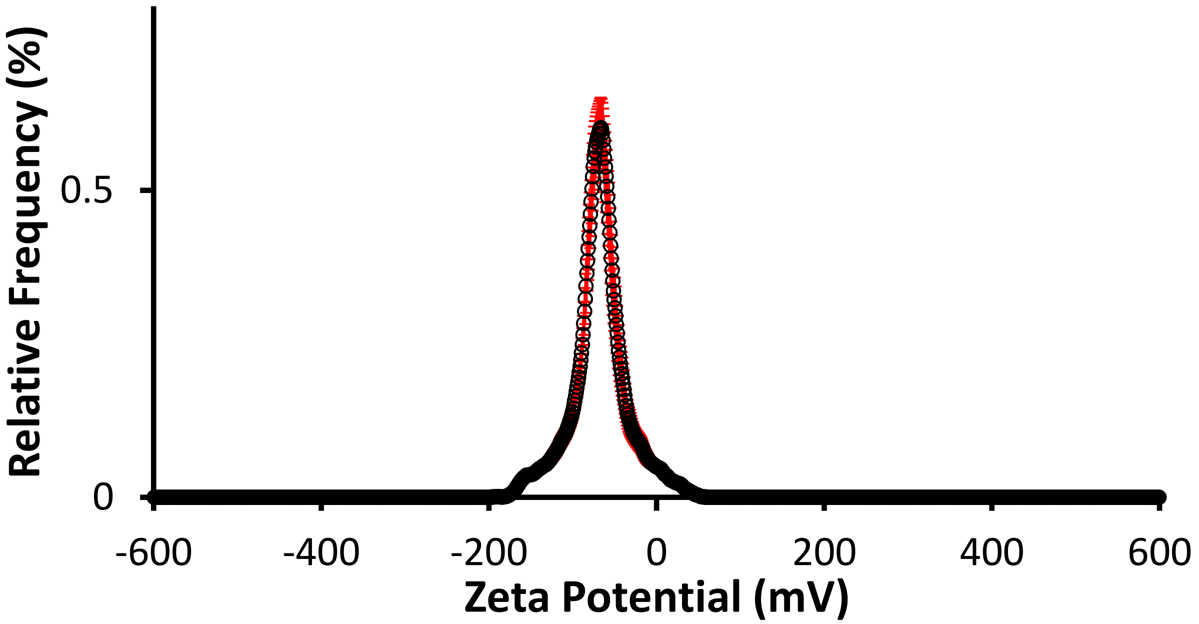

Figure S16 - The average zeta potential distribution calculated using 10 runs for compound **1** (0.56 mM) in an EtOH:H_2O (1:19) solution at 298 K. Average measurement value **-**67 mV.

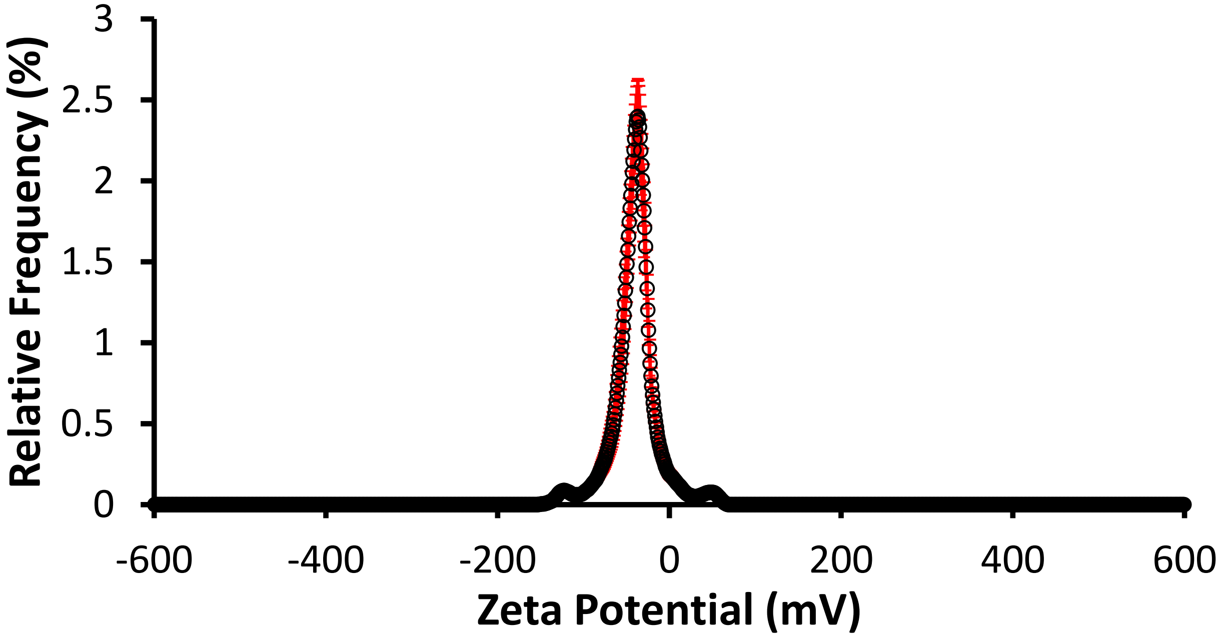

Figure S17 - The average zeta potential distribution calculated using 10 runs for compound **6** (0.56 mM) in an EtOH:H_2O (1:19) solution at 298 K. Average measurement value **-**44 mV.

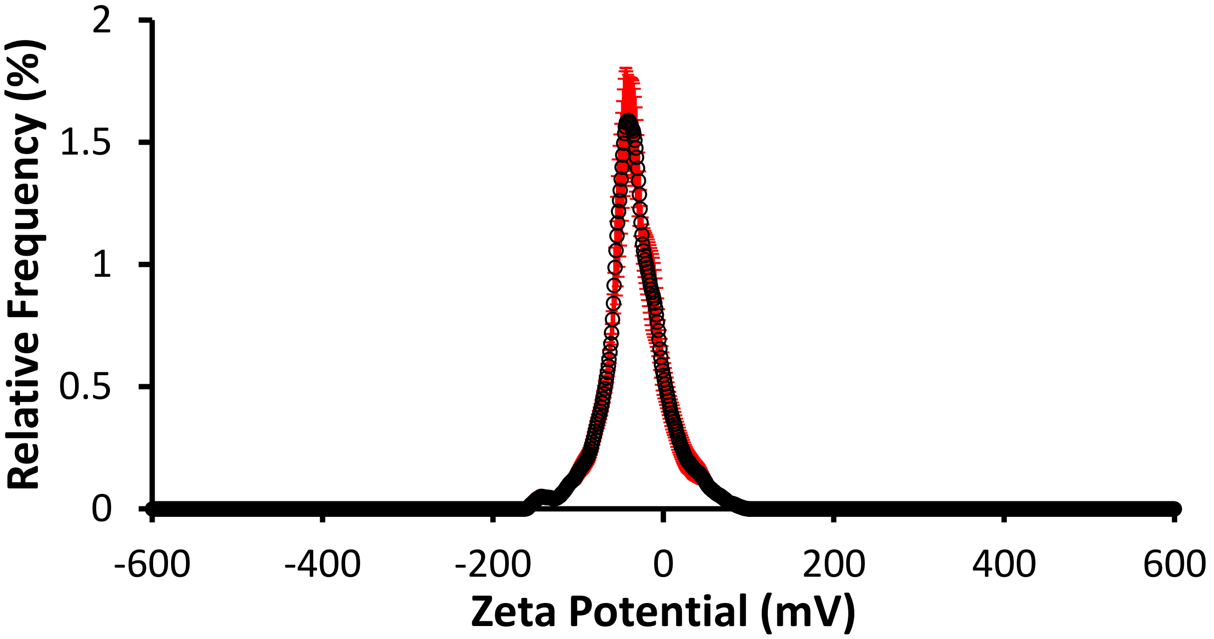

Figure S18 - The average zeta potential distribution calculated using 10 runs for a co-formulation of cisplatin (0.56 mM) and **1** (0.56 mM) in an EtOH:H_2O (1:19) solution at 298 K. Average measurement value -42 mV. A zeta potential value of -67 mV was obtained for compound **1** (0.56 mM) only.

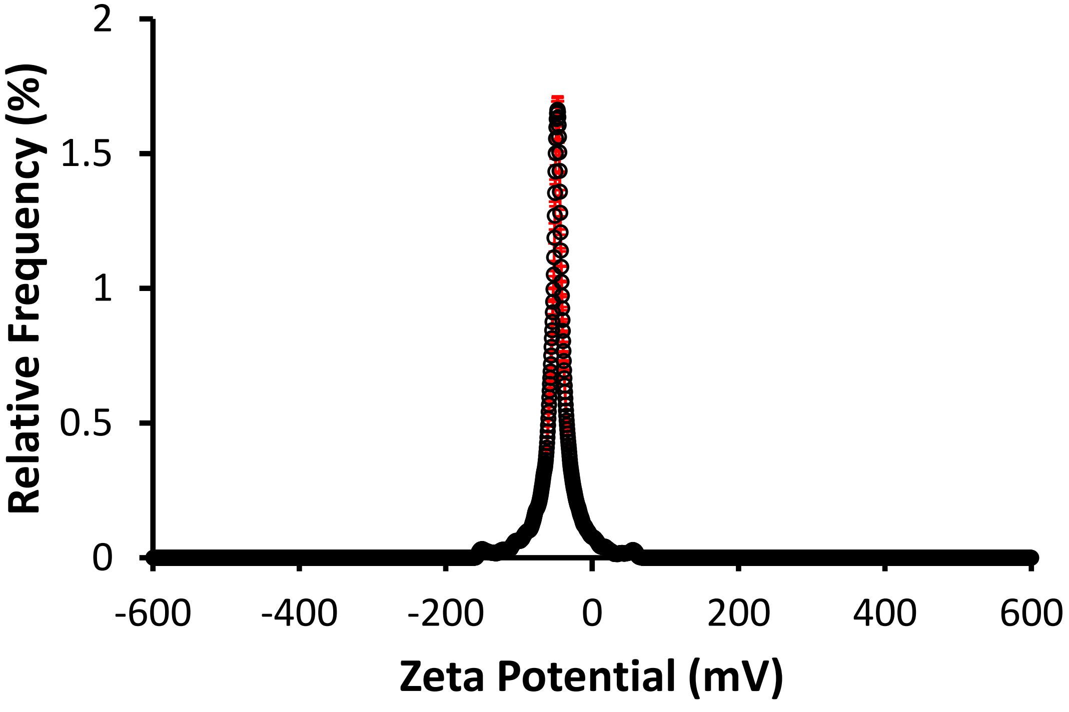

Figure S19 - The average zeta potential distribution calculated using 10 runs for a co-formulation of cisplatin (0.56 mM) and **6** (0.56 mM) in an EtOH:H_2O (1:19) solution at 298 K. Average measurement value -53 mV. A zeta potential value of -44 mV was obtained for compound **6** (0.56 mM) only.

Dynamic light scattering data

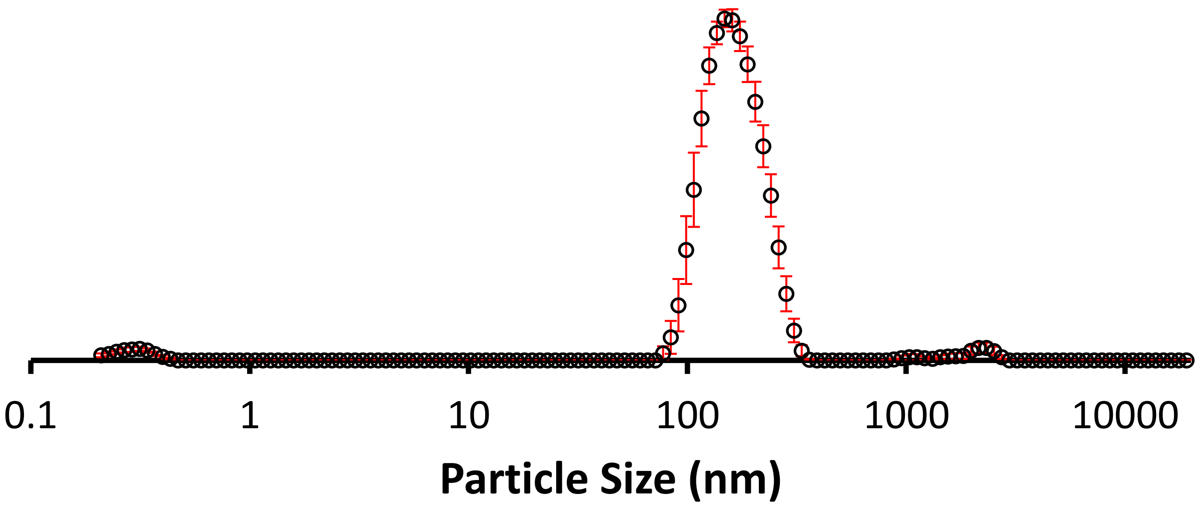

Figure S20 - The average intensity particle size distribution calculated using 9 DLS runs for a co-formulation of cisplatin (0.56 mM) and **1** (0.56 mM) in an EtOH:H_2O (1:19) solution at 298 K, with a peak maxima of 161 nm. A peak maxima of 142 nm was obtained for compound **1** only.[2]

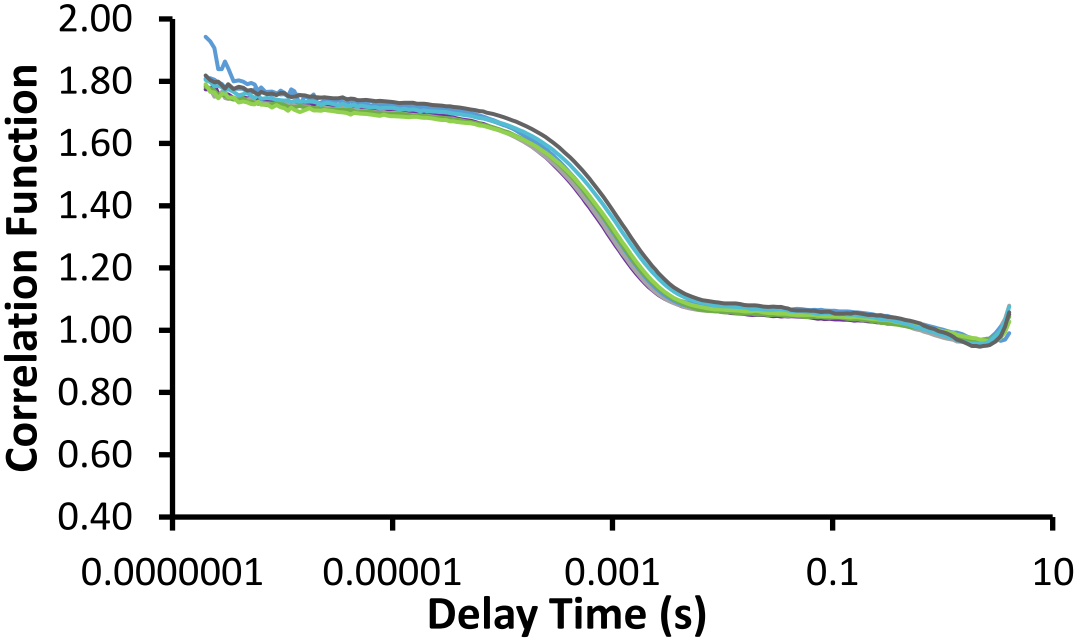

Figure S21 - Correlation function data for 9 DLS runs for a co-formulation of cisplatin (0.56 mM) and **1** (0.56 mM) in an EtOH:H_2O (1:19) solution at 298 K.

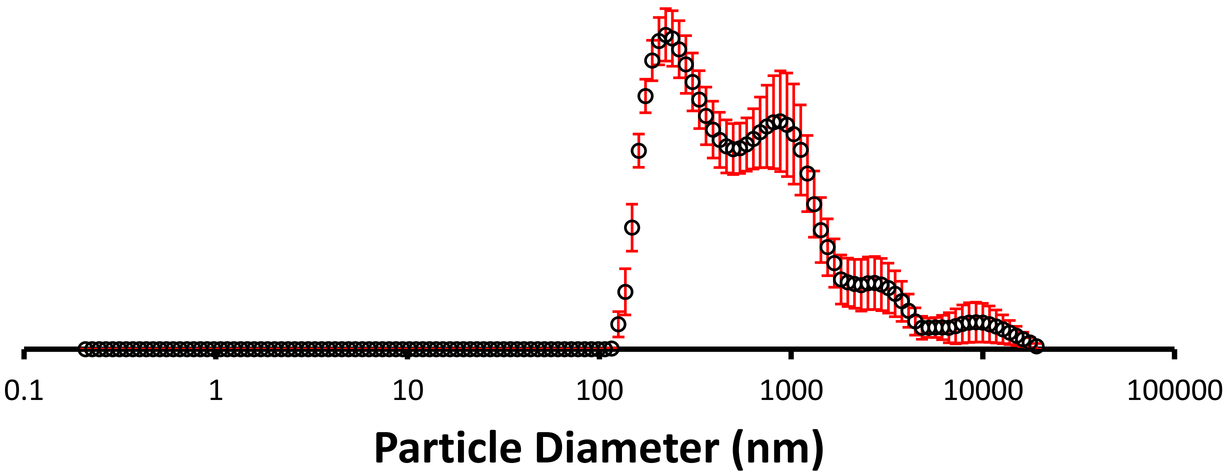

Figure S22 - The average intensity particle size distribution calculated using 9 DLS runs for a co-formulation of cisplatin (0.56 mM) and **6** (0.56 mM) in an EtOH:H_2O (1:19) solution at 298 K, with a peak maxima of 240 nm. A peak maxima of 300 nm was obtained for compound **6** only.[2]

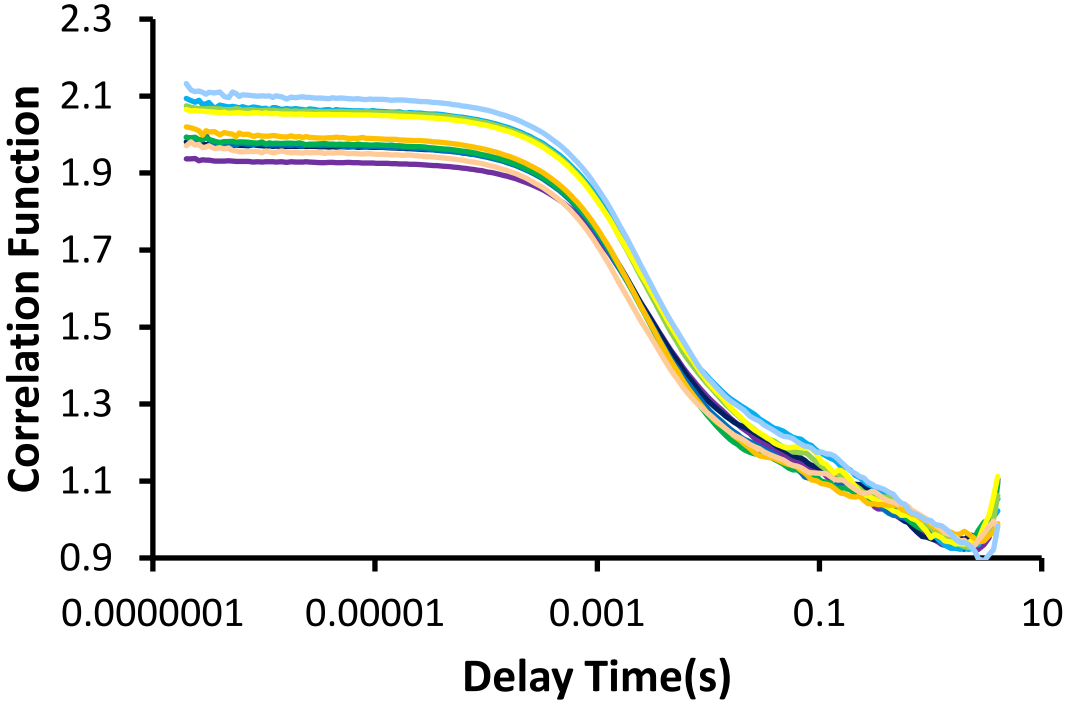

Figure S23 - Correlation function data for 9 DLS runs for a co-formulation of cisplatin (0.56 mM) and **6** (0.56 mM) in an EtOH:H_2O (1:19) solution at 298 K.

Surface Tension and Stability Data

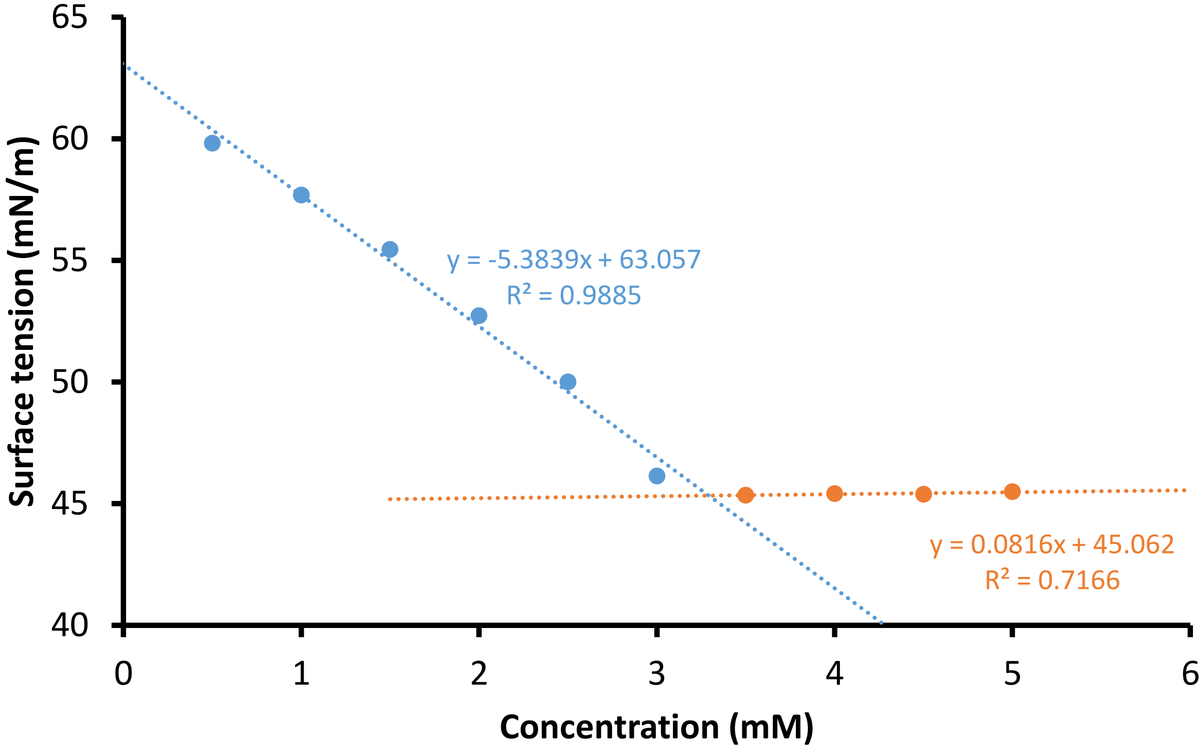

Figure S24 - Calculation of CMC (3.29 mM) for a 1:1 co-formulation of cisplatin and **1** in an EtOH: H_2O 1:19 mixture using surface tension measurements. A CMC of 10.40 mM was calculated for compound **1** only.[2]

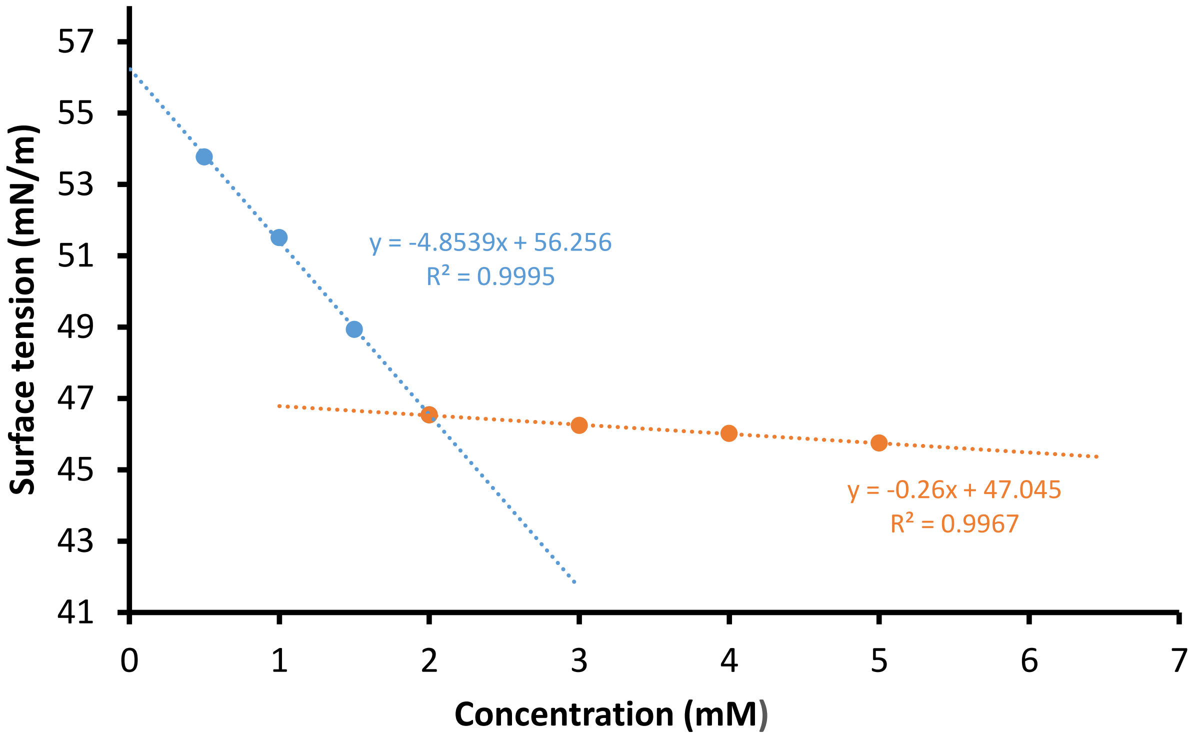

Figure S25 - Calculation of CMC (2.00 mM) for a 1:1 co-formulation of cisplatin and **6** in an EtOH: H_2O 1:19 mixture using surface tension measurements. A CMC of 0.50 mM was calculated for compound **6** only.[2]

Drug Combination Assay

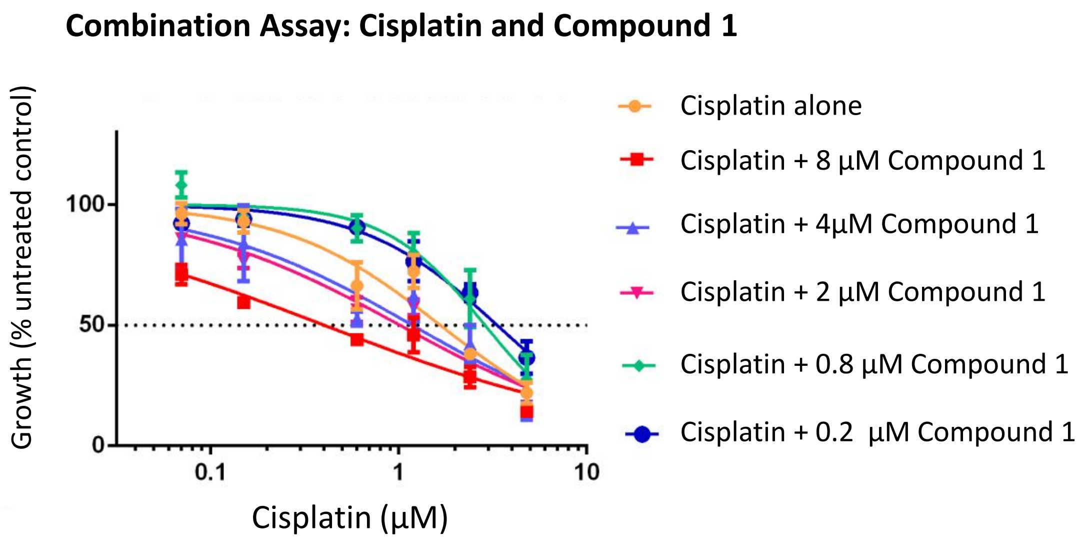

Figure S26 - Graph showing effects on A2780 cells of Cisplatin alone and when combined with Compound **1** at 0.2, 0.8, 2, 4 and 8 μM, corresponding to GI $_{0.25,\ 1,\ 2.5,.5,\ 10,}$ respectively, and incubated for 96 hours on cells followed by analysis as per SRB assay.. Data represents n=3 separate experiments.

Cell confluency assay

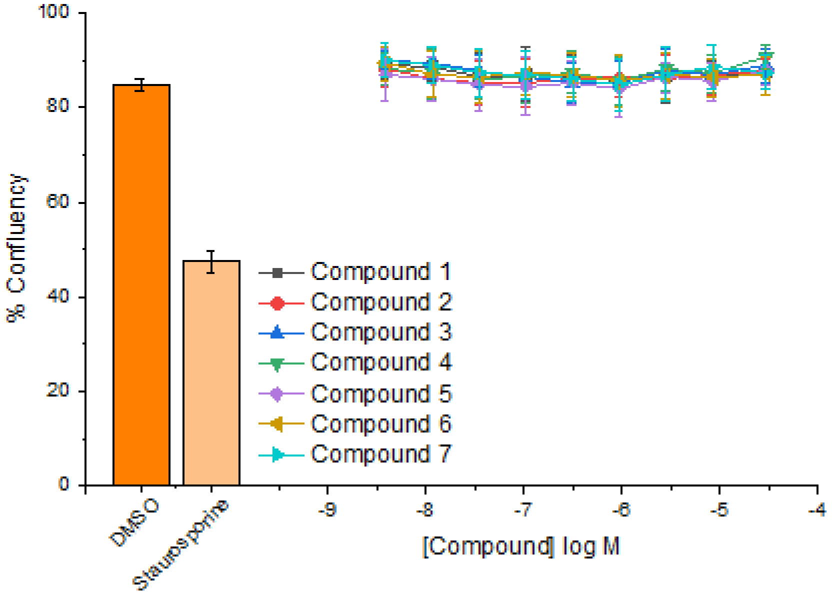

Figure S27 - Cell confluency of normal human dermal fibroblasts in the presence of **1-7**, a 0.3 % DMSO vehicle (as described) and a Staurosprine (500 nm) to demonstrate cytotoxicity. Cells were imaged using the IncuCyte S3 to quantify percentage confluency changes (using image analysis algorithms).

Cell viability assay

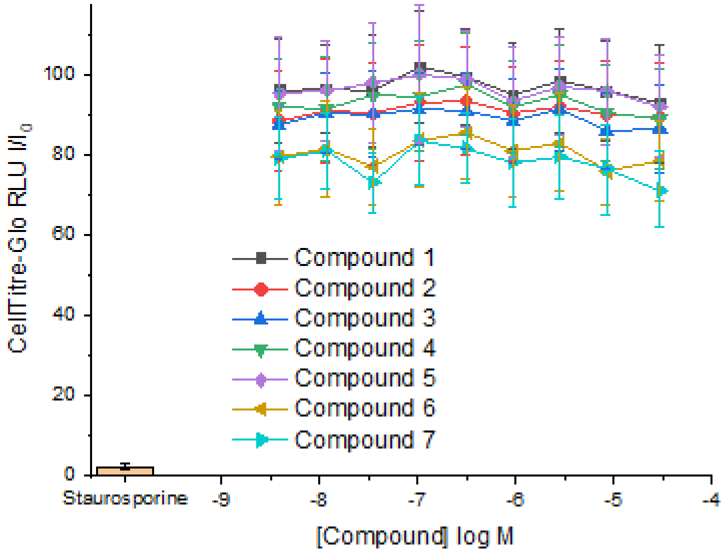

Figure S28 – Cell viability measured as a function of the relative intensity luminescence of normal human dermal fibroblasts treated with CellTitre-Glo reagent in the presence of **1-7** and staurosprine (500 nm) to demonstrate cytotoxicity, compared to a blank (previously described 0.3 % DMSO vehicle).

References

1. J. R. Hiscock, G. P. Bustone, B. Wilson, K. E. Belsey and L. R. Blackholly, *Soft Matter*, 2016, **12**, 4221-4228.
2. L. J. White, S. N. Tyuleva, B. Wilson, H. J. Shepherd, K. K. L. Ng, S. J. Holder, E. R. Clark and J. R. Hiscock, *Chem. Eur. J.*, 2018, **24**, 7761–7773.
3. L. R. Blackholly, H. J. Shepherd and J. R. Hiscock, *CrystEngComm.*, 2016, **18**, 7021-7028
4. T. C. Chou and P. Talalay, *Adv. Enzyme Regul.* 1984, **22**, 27-55.
5. M. M. Bradford, *Anal. Biochem.,* 1976, **72**, 248–254.

‡: Trevor.Askwith@Domainex.co.uk
